# Content, Structure, and Delivery Characteristics of Yoga Interventions for Managing Hypertension: A Systematic Review and Meta-Analysis of Randomized Controlled Trials

**DOI:** 10.3389/fpubh.2022.846231

**Published:** 2022-03-28

**Authors:** Gamze Nalbant, Zeinab M. Hassanein, Sarah Lewis, Kaushik Chattopadhyay

**Affiliations:** ^1^Lifespan and Population Health Academic Unit, School of Medicine, University of Nottingham, Nottingham, United Kingdom; ^2^The Nottingham Centre for Evidence-Based Healthcare, A JBI Centre of Excellence, Nottingham, United Kingdom

**Keywords:** hypertension, management, yoga, systematic review, meta-analysis

## Abstract

**Objectives:**

This systematic review aimed to synthesize the content, structure, and delivery characteristics of effective yoga interventions used for managing hypertension and to compare these characteristics with ineffective interventions.

**Design and Method:**

The JBI and the PRISMA guidelines were followed in this systematic review. RCTs conducted among hypertensive adults were included. RCTs reporting at least one of the major components of yoga (i.e., asana, pranayama, and dhyana and relaxation practices) and comparing them with no intervention or any intervention were eligible. Sixteen databases were searched for published and unpublished studies without any date and language restrictions till March 15, 2021.

**Results:**

The literature search yielded 13,130 records. 34 RCTs (evaluating 38 yoga interventions) met the inclusion criteria. Overall, included studies had low methodological quality mostly due to inadequate reporting. Yoga reduced SBP and DBP compared to a control intervention (MD −6.49 and −2.78; 95CI% −8.94– −4.04 and −4.11– −1.45, respectively). Eighteen, 14 and 20 interventions were effective in improving SBP, DBP, or either, respectively. 13 out of 20 effective interventions incorporated all the 3 major components of yoga and allocated similar durations to each component whereas ineffective interventions were more focused on the asana and duration of asana practice was longer. The most common duration and frequency of effective interventions were 45 min/session (in 5 interventions), 7 days/week (in 5 interventions), and 12 weeks (in 11 interventions) whereas the most common session frequency was 2 days a week (in 7 interventions) in ineffective interventions. Effective interventions were mostly center-based (in 15 interventions) and supervised (in 16 interventions) and this was similar with ineffective interventions.

**Conclusion:**

Despite the low quality and heterogeneity of included studies, our findings suggest yoga interventions may effectively manage hypertension. The differences between the effective and ineffective interventions suggest that effective yoga interventions mostly incorporated asana, pranayama, and dhyana and relaxation practices and they had a balance between these three components and included regular practice. They were mostly delivered in a center and under supervision. Future studies should consider developing and evaluating an intervention for managing hypertension using the synthesized findings of the effective interventions in this review.

**Systematic Review Registration:**

[PROSPERO], identifier [CRD42019139404].

## Introduction

The number of people with hypertension in the world has significantly increased in the last four decades (1). Current estimates suggest that the global prevalence of hypertension is around one billion (1). If this trend continues, by 2025, ~1.56 billion people aged over 20 years are expected to have hypertension (2, 3). Hypertension has a close link with serious health problems such as cardiovascular disease, cerebrovascular disease, and kidney disease; therefore, it is considered a significant global threat (4). Hypertension also imposes a great economic burden (5); including both direct medical costs for hypertension treatment and the costs of comorbidities attributable to hypertension and indirect costs such as productivity loss due to absence from work (6).

Hypertension is characterized by consistently high arterial blood pressure, and the International Society of Hypertension and the American Society of Hypertension (ISH/ASH) classifies hypertension into three stages as prehypertension (SBP: 120–139 mmHg, DBP: 80–89 mmHg), stage 1 hypertension (SBP: 140–59 mmHg, DBP: 90–99 mmHg) and stage 2 hypertension (SBP ≥160, DBP ≥100 mmHg) (7). Current hypertension management strategies aim to reduce blood pressure and include lifestyle modification, mainly dietary changes, physical activity, and stress reduction, in addition to pharmacological treatment (8). The current hypertension management strategies achieved a substantial improvement in the awareness, diagnosis, and management of hypertension (1). However, the World Health Organization (WHO) 2013 report emphasized the importance of ‘thinking outside the box' for creative ways to further improve the management of hypertension (9). Yoga, as a non-pharmacological intervention, could be one such additional way to further improve the management of hypertension.

Yoga, ancient Indian philosophy and way of life, originated in the Indian subcontinent over 5,000 years ago and is being used as a method of improving health and wellbeing (10). There are different branches of yoga, each with a different emphasis on and approach to practice; Hatha yoga, an overarching term for physically-based yoga styles such as Iyengar, Asthanga, and Vinyasa, is the most commonly practiced style (11–13). Yoga, as explained by Patanjali, has eight components which are ethical standards (yama), self-discipline (niyama), yogic poses (asana), breathing practices (pranayama), withdrawal of the senses (pratyahara), concentration (dharana), meditation (dhyana) and relaxation practices, and transcendence (samadhi) (14, 15).

The beneficial effects of yoga on hypertension may occur through its blood pressure-lowering effect (11), as well as its effects on physical activity, stress reduction, and lifestyle (16–19). The exact mechanism of yoga for lowering blood pressure is not known yet but proposed physiological mechanisms include an increase in parasympathetic activity, possibly due to vagal stimulation, the suppression of the hypothalamic-pituitary-adrenal axis, and reduction in the activity and reactivity of the sympathetic nervous system (20–23). Yoga, as physical activity, can satisfy the recommended levels of physical activity for people with hypertension. For example, one study found that an hour of Ashtanga yoga satisfies the moderate levels of physical intensity (16) and another study found that 1.5 h of Vinyasa yoga is higher than moderate physical activity requirements (24). 2020 ISH Global Hypertension Practice Guidelines also suggest yoga as an aerobic form of physical activity under lifestyle modification (8). Yoga has been shown to improve stress levels (18, 25), which is associated with high blood pressure (8). In addition, yoga focuses on a healthier and mindful lifestyle, which supports people for a healthier diet, and abstinence from smoking and alcohol (26–28).

Several systematic reviews have evaluated and synthesized evidence on the effectiveness of yoga interventions for managing hypertension (10, 20, 29–35). Previous reviews showed that yoga interventions significantly reduced SBP and DBP (10, 32, 33). Six of these reviews, despite claiming to include studies conducted on patients with hypertension and yoga interventions, included studies where not all the participants were hypertensive (10, 29, 30, 34), yoga interventions were part of multimodal interventions (10, 29, 35) or interventions other than yoga were assessed (30, 33). In addition, though these systematic reviews suggested that yoga might be effective in hypertension management, they were inconclusive. It is also hard to select one yoga intervention over the other because of their heterogeneous content, structure, and delivery characteristics, which are important aspects of yoga interventions. To evaluate yoga's effectiveness, there is a need to summarize and synthesize potentially effective aspects of yoga interventions. Only one systematic review (29), described the content and structure of yoga interventions used in each study; however, this review did not synthesize these aspects of effective interventions. Thus, the objective of this systematic review was to synthesize the content, structure, and delivery characteristics of effective yoga interventions used for managing hypertension and to compare these characteristics with ineffective interventions.

## Materials and Methods

This systematic review was conducted in accordance with the JBI methodology for systematic reviews of effectiveness and the Preferred Reporting Items for Systematic Reviews and Meta-Analyses (PRISMA) guidelines (36, 37). The systematic review protocol has been published elsewhere (38) and it was registered with PROSPERO (CRD42019139404). Two independent reviewers were involved throughout the process and any disagreements arising between reviewers were resolved through discussion. If consensus was not reached, a third reviewer was involved.

### Inclusion Criteria

#### Population

This systematic review included studies conducted among adults (≥18 years) diagnosed with hypertension. The International Society of Hypertension and the American Society of Hypertension (ISH/ASH) classifies hypertension into three stages: prehypertension (SBP: 120–139 mmHg, DBP: 80–89 mmHg), stage 1 hypertension (SBP: 140–59 mmHg, DBP: 90–99 mmHg) and stage 2 hypertension (SBP ≥160, DBP ≥100 mmHg) (7). Studies in line with this classification were eligible. In accordance with the ISH/ASH guideline, studies conducted among participants with isolated systolic or diastolic hypertension were also eligible. A study that included adults and non-adults was included if the participants' mean age was ≥18 and results were stratified by age so that relevant data could be extracted.

#### Intervention

Studies reporting at least one of the major components of yoga (i.e., asana, pranayama, and dhyana and relaxation practice) were included. Studies on multimodal interventions that included yoga among others were excluded if the relevant data were not possible to extract. Studies were excluded if they did not explicitly label the intervention as yoga. Studies examining acute effects of yoga interventions on SBP and/or DBP following a single session were included as well as long-term studies.

#### Comparator

Studies comparing yoga interventions with no intervention, sham intervention, any non-pharmaceutical intervention (such as diet, exercise, or yoga), or pharmaceutical intervention (such as antihypertensive drugs) were included in this systematic review. Studies allowing co-interventions were included (i.e., studies allowing participants to continue their individual treatment were included as long as all the eligible study groups were allowed to do so).

#### Outcome

This systematic review included studies that assessed SBP and DBP as outcomes.

#### Study Design

Only RCTs were included in this systematic review.

### Data Sources and Search Strategy

Studies were identified using a three-step search strategy. First, an initial limited search was conducted in the MEDLINE database using keywords such as yoga, hypertension, and RCT. The search results were inspected to ensure that the relevant articles were identified. The text words contained in the titles and abstracts of relevant articles and the index terms used to describe the articles were used to develop a search strategy for MEDLINE in consultation with an information specialist/librarian. Second, the search strategy was adapted for other databases in consultation with the information specialist/librarian, and a systematic search across all included databases was undertaken to identify published and unpublished studies. Third, the reference list of previous systematic reviews and studies included in the systematic review were screened for additional studies. The search strategies are reported in the Supplementary File.

The following 16 databases were searched, without date and language restrictions, till March 15, 2021: MEDLINE (from 1946, Ovid), Embase (from 1947, Ovid), CINAHL (from 1937, EBSCO), PsycINFO (from 1806, Ovid), Allied and Complementary Medicine (AMED, from 1985, Ovid), Web of Science (from 1900), Cochrane Central Register of Controlled Trials (CENTRAL, from 1996), Turning Research Into Practice (TRIP, from 1997), AYUSH Research Portal, A Bibliography of Indian Medicine (ABIM), Digital Helpline for Ayurveda Research Articles (DHARA), CAM-QUEST and Directory of Open Access Journals (DOAJ). The search for unpublished studies included OpenGrey (from 1997), EthOS (from 1925), and ProQuest Dissertations and Theses (from 1980, ProQuest).

### Screening and Full-Text Reading

Following the search, all identified citations were collated and uploaded into Endnote X8.2 (Clarivate Analytics, PA, USA) (39) and duplicates were removed. The remaining records were then imported into Rayyan (Qatar Computing Research Institute [Data Analytics], Doha, Qatar) (40), a web application, to facilitate the title and abstract screening process. Titles and abstracts were independently screened for eligibility using the inclusion criteria by two reviewers. Studies identified as potentially eligible or those without an abstract were retrieved in full. The full-text of these studies was assessed in detail against the inclusion criteria by the two independent reviewers. Full-text studies that did not meet the inclusion criteria were excluded and the reasons for exclusion were reported. Any disagreements that arose between the reviewers were resolved through discussion. If consensus was not reached, then a third reviewer was involved.

### Methodological Quality Assessment

Included studies were critically assessed using the standardized JBI critical appraisal tool for RCTs (41). This tool has 13 criteria that can be scored as being met (yes), not met (no), unclear, or not applicable (n/a). One of the criteria was about the blinding of intervention deliverers, which was not possible with yoga interventions and accepted as not applicable. The quality of the individual studies was determined as high quality if 8 or more of criteria scored yes, moderate quality if 5–7 of criteria scored yes, and low quality if 4 or less of criteria scored yes. Two independent reviewers were involved throughout the process and any disagreements arising between reviewers were resolved through discussion. If consensus was not reached, a third reviewer was involved. All studies, regardless of their methodological quality, were included in the review.

### Data Extraction

Data were extracted using a predeveloped and piloted data extraction form. The data extracted included the characteristics of the studies: author, year of publication, country, final follow-up, participant characteristics (e.g., age, sex, stage of hypertension, medication use, comorbidities, and previous experience of yoga), sample size, intervention and comparator, study outcomes (e.g., blood pressure and adverse events), final follow-up (in weeks), lost to follow-up, and SBP and DBP data extraction time-point (in weeks). In addition, the characteristics of yoga interventions were extracted: intervention development, structure (e.g., session duration, frequency, and intervention duration), delivery characteristics (e.g., the context of the intervention, strategies used to enhance intervention uptake and adherence, and characteristics of yoga instructors), and content (e.g., asana, pranayama, and dhyana and relaxation practices).

The beneficial effects of treatment for most patients with hypertension would be reached within 8 weeks (7, 42). Therefore, the authors extracted the 8-week time point data. Where this time point data was not reported or multiple time points were reported, data from the time point closest to 8 weeks from the start of the intervention were extracted. Intention-to-treat (ITT) data were preferred compared to per-protocol data and post-intervention data preferred over the change from baseline data (i.e., post-intervention score—baseline score). Mean change from baseline was used if only change scores were reported and if there was a significant difference between groups at baseline (≥5 mmHg). However, if studies with a significant difference at baseline did not report change scores, post-intervention data were used, and the imbalance was noted in the risk of bias assessment.

SBP and DBP are continuous data and so mean and SD were extracted. Where no SDs were available, they were calculated from SE, CI, or interquartile ranges, and where no mean scores were available; mean was assumed to be equal to median. In case of missing or unclear data, authors were contacted by e-mail (two times per author).

### Data Synthesis

A tabular and narrative format was used to synthesize results. Some yoga interventions included only one component whereas others incorporated two or three components; therefore, yoga interventions were divided as those incorporating three components of yoga (i.e., yoga interventions incorporating asana, pranayama and dhyana, and relaxation practices), those combining any two components of yoga (e.g., asana and pranayama) and only pranayama interventions. To determine the effectiveness of the studies, a meta-analysis using Review Manager 5.4.1 (43) was conducted as many included RCTs used inappropriate analysis methods to decide the effectiveness of the intervention. First, all yoga interventions were compared with all control groups to show the overall effectiveness of yoga interventions. Then, subgroup analyses were done according to the type of yoga interventions and type of comparators separately for SBP and DBP. Yoga interventions were divided as those incorporating three components of yoga (i.e., yoga interventions incorporating asana, pranayama, and dhyana and relaxation practices), those combining any two components of yoga (e.g., asana and pranayama), and only pranayama interventions. The plan was to divide comparator groups into three as no-intervention, non-pharmaceutical intervention, and pharmaceutical intervention. However, only one study had pharmaceutical intervention as a comparator group. Therefore, comparator groups were divided into two subgroups as no-intervention and non-pharmaceutical intervention. Studies allowing co-medication for both intervention and comparator groups, with no additional intervention, comparator groups were evaluated as no-intervention. All active comparator groups were evaluated as non-pharmaceutical interventions. If yoga interventions were compared with both no intervention and non-pharmaceutical intervention groups within the same study, they were considered separately. Yoga interventions shorter than 8 weeks were separated from the long-term studies in the meta-analysis.

In the meta-analysis, random effects were used due to the heterogeneous nature of the interventions. Statistical heterogeneity was assessed in the meta-analysis using the I^2^ and heterogeneity was considered substantial if I^2^ > 50%. Where there was a sufficient number of studies included in the meta-analysis (at least 10), funnel plots were generated to assess for publication bias.

## Results

### Study Selection

**Figure 1:**
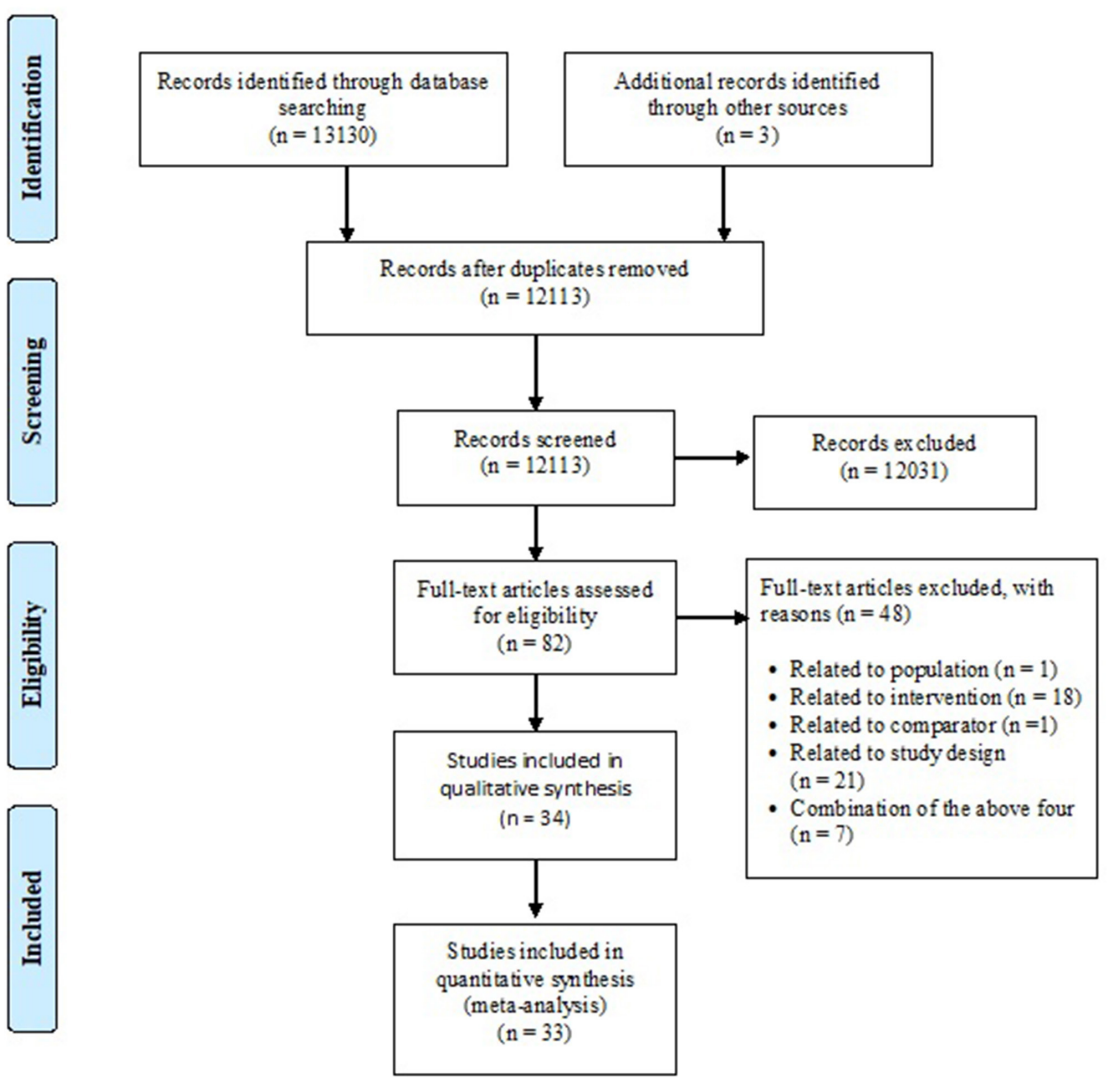
PRISMA flow diagram.

Thirteen thousand one hundred thirty records were identified through the literature search. After the removal of duplicate records and title and abstract screening, 82 studies were retrieved for full-text screening. Thirty-four RCTs met the inclusion criteria and were included in this systematic review (44–77). (See Figure 1 for PRISMA flowchart) (37) The list of studies found ineligible following full-text review and ongoing RCTs identified from trial registries are presented in the Supplementary File.

### Description of the Included Studies

See Supplementary Table 1 for the characteristics of the included studies. Thirty-four RCTs met the inclusion criteria. Fifteen studies reported the number of people invited and participated. In total, 5,083 people were invited and 3,125 participated in these studies. Twenty-one RCTs were conducted in India (*n* = 1,694 participants), 4 in the USA (*n* = 382) (45, 47, 53, 59), 2 in Germany (*n* = 415) (46, 67), 2 in Nepal (*n* = 135) (61, 77) and 1 each in Sweden (*n* = 191) (49), Thailand (*n* = 61) (63), Spain (*n* = 100) (64), Hong-Kong (*n* = 97) (68) and Brazil (*n* = 50) (72). Studies recruited participants who were between 18 and 70 years old. Four RCTs recruited only male participants (50, 54, 67, 70). Two recruited only female participants (72, 74) and the sex of the participants was unclear in 3 RCTs (44, 51, 69). Nineteen out of 34 studies did not specify the stages of hypertension. Four studies included only participants with prehypertension, 4 included only participants with stage I hypertension, 5 included participants with prehypertension and stage I hypertension, 1 included participants with stage I and II hypertension and 1 included participants at any stage of hypertension. All the participants were on antihypertensive medication in 12 RCTs, some of the participants were on antihypertensive medication in 5 RCTs and none of the participants was on antihypertensive medication in 7 RCTs. Ten studies did not provide information if participants were on antihypertensive medication. Additional health conditions of the participants included postmenopause (*n* = 2) (72, 74), type 2 diabetes mellitus (T2DM) (*n* = 2) (58, 71) and metabolic syndrome (MetS) (*n* = 1) (68). Previous experience of yoga practice among participants varied across the RCTs and 17 studies recruited yoga-naïve participants whereas 2 studies recruited participants with experience of practicing yoga.

### Description of the Included Interventions and Comparators

See Supplementary Table 2 for the details of the yoga interventions (e.g., content, structure, and delivery characteristics). Six studies included more than one yoga group and these were considered as separate interventions unless they used the same yogic practice with varying context and delivery characteristics. Consequently, 38 yoga interventions within 34 studies were identified. Of the 38 interventions, 21 incorporated all the three components of yoga (asana, pranayama, and dhyana and relaxation practice); 11 included only pranayama; 3 included pranayama, and dhyana and relaxation practice; 2 included asana and pranayama and one included asana, and dhyana and relaxation practice.

Yoga interventions were compared with a variety of comparators such as no intervention, no additional intervention (where both intervention and control groups received the same intervention e.g., antihypertensive/antidiabetic treatment and lifestyle modification training), lifestyle modification training, exercise, acupuncture, Buteyko breathing, sham breathing, and sham relaxation. Four studies compared yoga intervention with more than one comparator and 38 comparators within 34 studies were identified.

### Methodological Quality of the Studies

Overall, the studies were assessed as being of low quality (See Supplementary Table 3). The total number of “yes” answers was 7 or more in 6 studies, was 6 in 2 studies, was 5 in 8 studies, and was 4 or less in 18 studies. Even though the design and conduct of studies appeared to be good enough, the methodology was not adequately reported and this resulted in poor scoring. There were poor methodological quality studies as well. Some of the major issues in these studies were: (i) inadequate reporting of the randomization process, of the allocation concealment process, of the blinding of participants and outcome assessors, of whether the study arms were treated identically other than the intervention of interest or not, of the measurement process of outcomes (including adverse events); (ii) imbalance between the treatment groups at baseline; (iii) describing the differences between study arms in terms of their follow up but not analyzing these; (iv) not performing ITT analysis; (v) errors/issues in the sample size calculation and reporting; and (vi) errors/issues in data analysis and reporting (e.g., pre-post analysis and not between the groups).

### Adverse Events

Adverse events were not reported in 26 RCTs and 6 RCTs reported that no adverse events occurred. 2 RCTs reported adverse events but only one of these studies provided details (46, 59). The study (46) reported that adverse events occurred in yoga and comparator groups but none of these adverse events was judged to be directly related to the yoga intervention.

### Publication Bias

Funnel plots were generated for yoga intervention vs. control (Supplementary Figures 1, 2) and asana, pranayama, and dhyana and relaxation practice vs. no intervention (Supplementary Figures 3, 4). Funnel plots showed little evidence of publication bias. Meta-analysis including all the yoga interventions showed substantial heterogeneity for SBP (I^2^ = 88%) and DBP (I^2^ = 85%). Subgroup analyses could not reduce heterogeneity substantially. Only two subgroup analyses comparing pranayama with no intervention and non-pharmaceutical interventions for DBP showed no heterogeneity.

### Meta-Analysis to Determine Study Effectiveness

**Figure 2:**
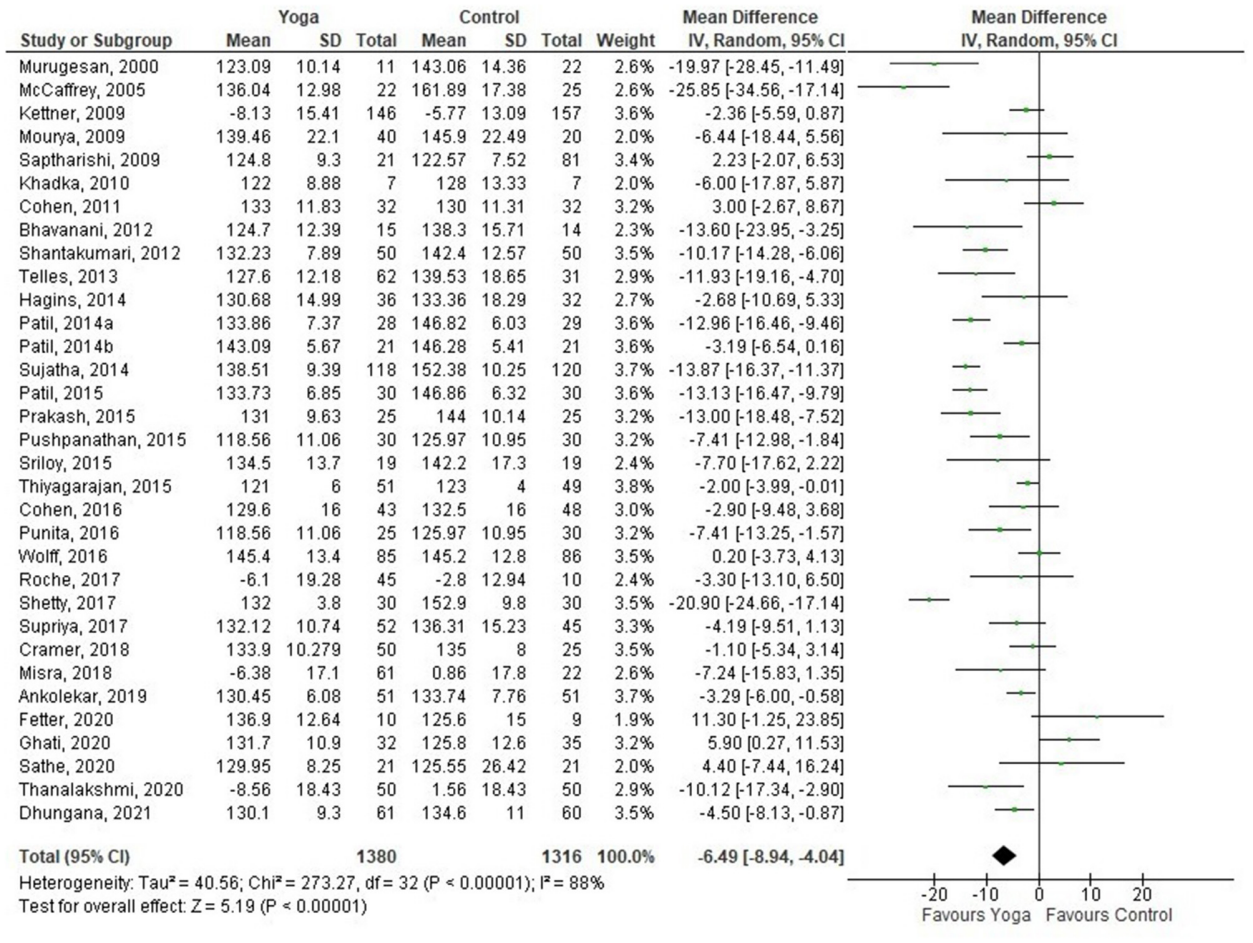
Yoga vs. control (SBP).

**Figure 3:**
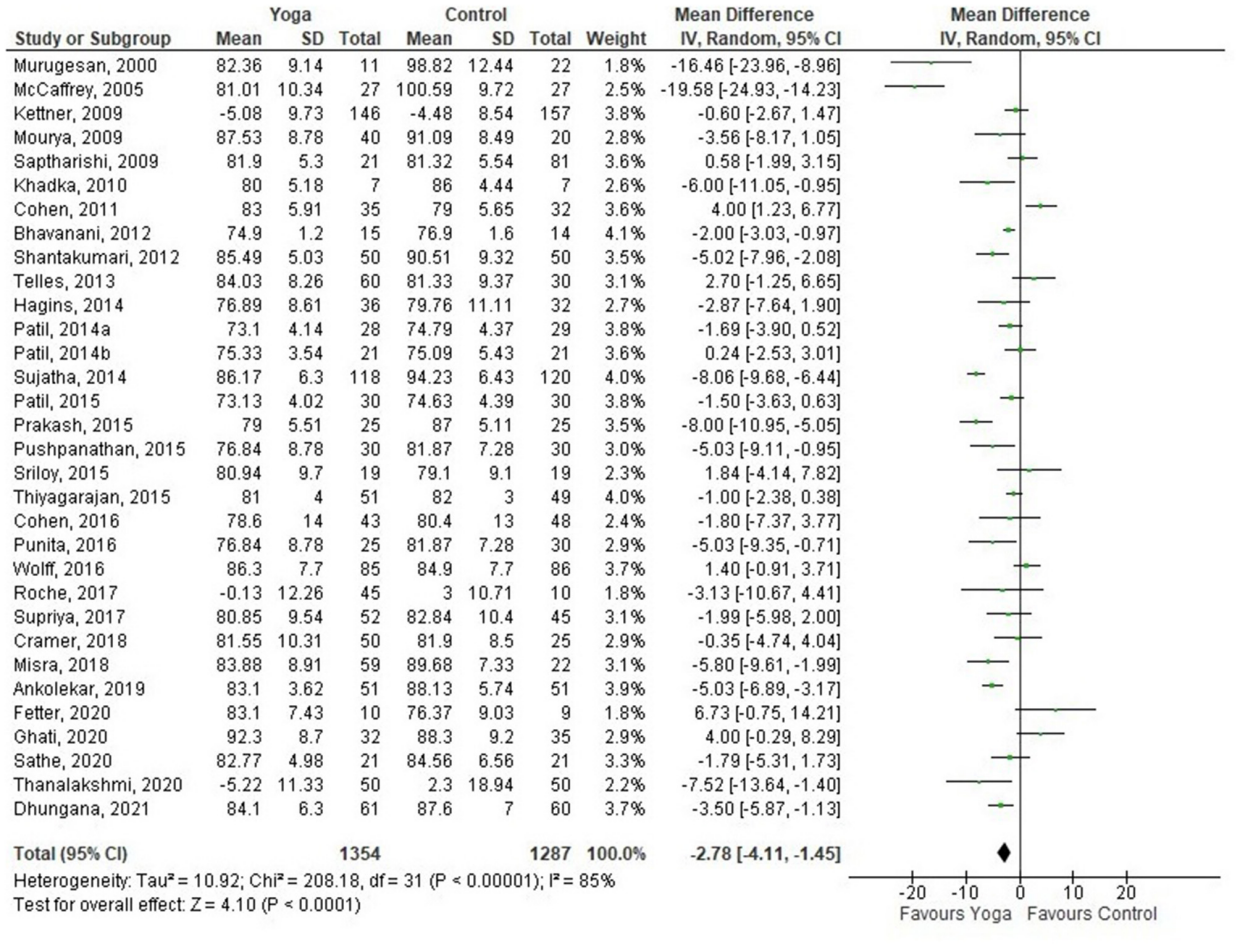
Yoga vs. control (DBP).

Thirty-three out of 34 studies were included in the meta-analysis, comparing yoga with any type of comparators. One of the studies (74) was not included as it compared a yoga intervention with another yoga intervention. The overall effect on SBP and DBP favored yoga interventions compared to comparators (mean difference (MD) −6.49 mmHg; 95% CI −8.94– −4.04; −2.78 mmHg; −4.11– −1.45, respectively) (Figures 2, 3). Of the studies included in the meta-analysis, 16, 13, and 18 interventions were effective in improving SBP, DBP, or either, respectively.

**Figure 4:**
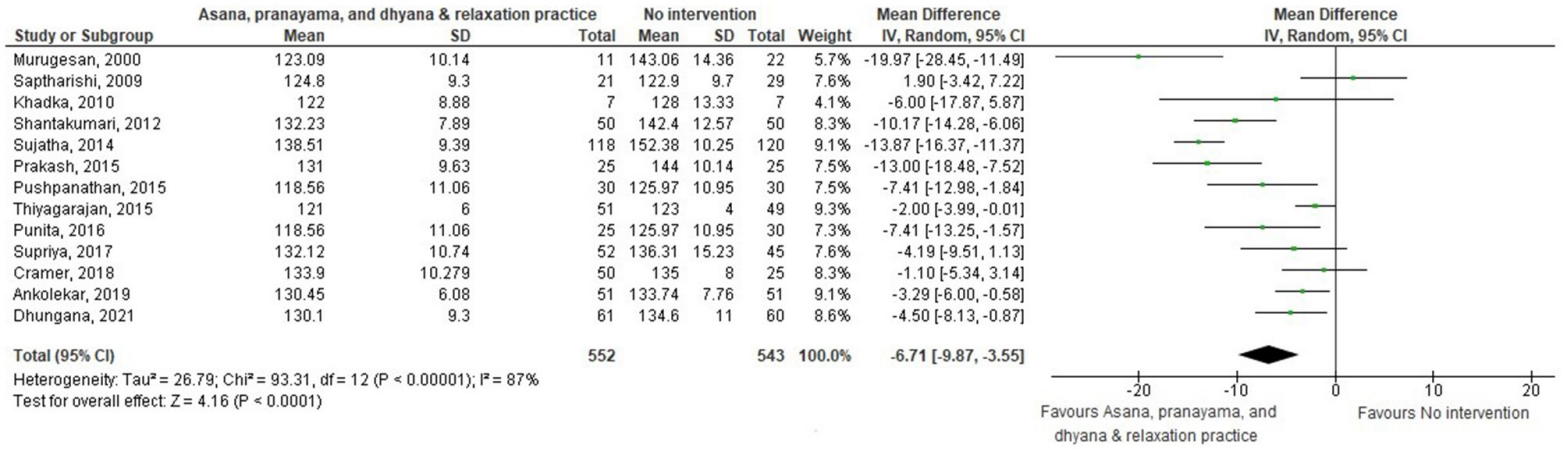
Asana, pranayama, and dhyana and relaxation practice vs. no intervention (SBP).

**Figure 5:**
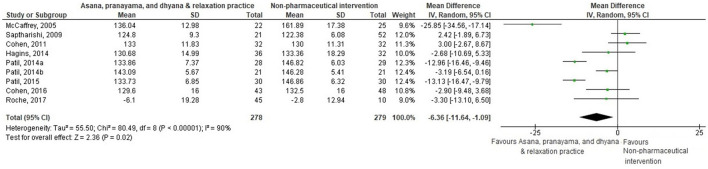
Asana, pranayama and dhyana and relaxation practice vs. non-pharmaceutical intervention (SBP).

**Figure 6:**
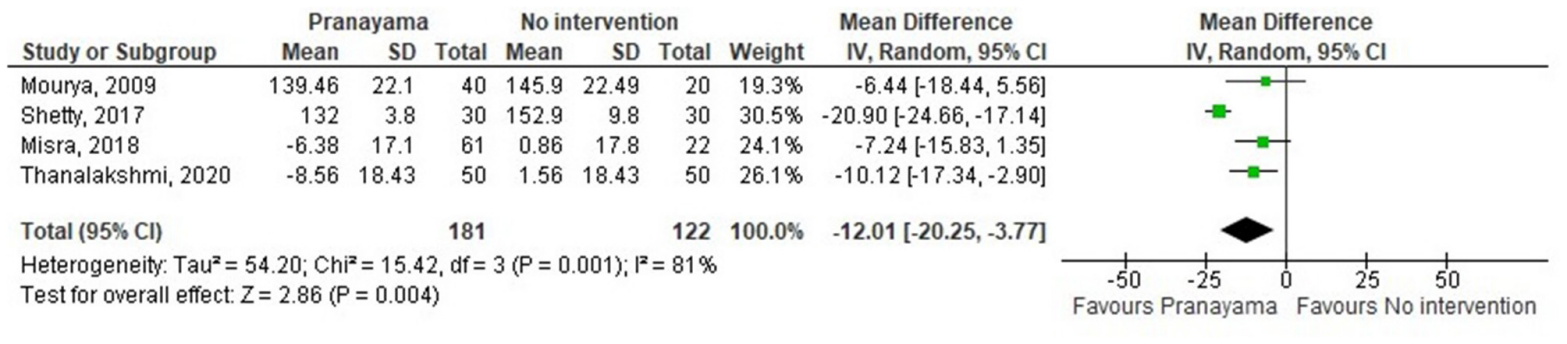
Pranayama vs. no intervention (SBP).

**Figure 7:**
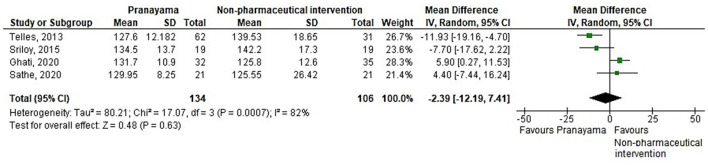
Pranayama vs. non-pharmaceutical intervention (SBP).

Thirty-eight comparisons were made in subgroup analyses, in total. In subgroup analyses, 18, 14, and 20 out of 38 yoga interventions showed effectiveness in reducing SBP, DBP, or either, respectively. All subgroup comparisons favored yoga interventions except for pranayama vs. non-pharmaceutical intervention in reducing SBP. Asana, pranayama, and dhyana and relaxation practice was more effective in reducing SBP compared to no intervention (−6.71 mmHg; −9.87– −3.55) and non-pharmaceutical interventions (−6.36 mmHg; −11.64– −1.09) (Figures 4, 5). Pranayama was more effective in reducing SBP compared to no intervention (−12.01 mmHg; −20.25– −3.77) but not compared to non-pharmaceutical intervention (−2.39 mmHg; −12.19–7.41) (Figures 6, 7).

**Figure 8:**
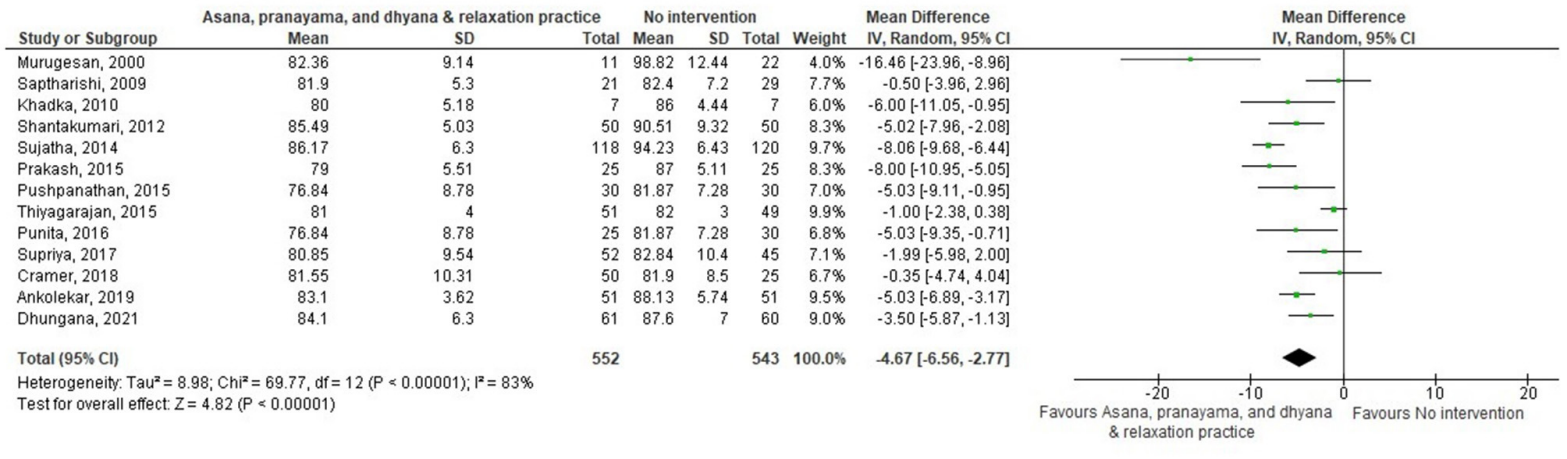
Asana, pranayama and dhyana and relaxation practice vs. no intervention (DBP).

**Figure 9:**
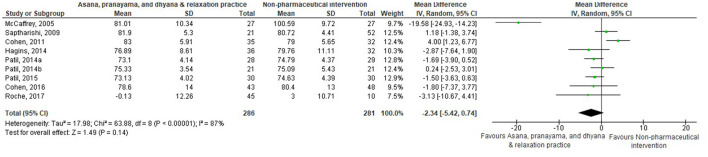
Asana, pranayama and dhyana and relaxation practice vs. non-pharmaceutical intervention (DBP).

**Figure 10:**
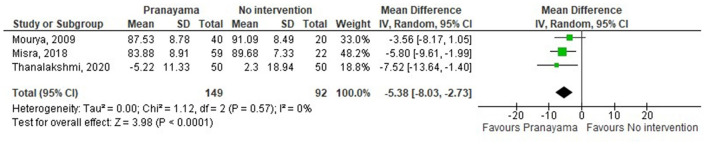
Pranayama vs. no intervention (DBP).

**Figure 11:**
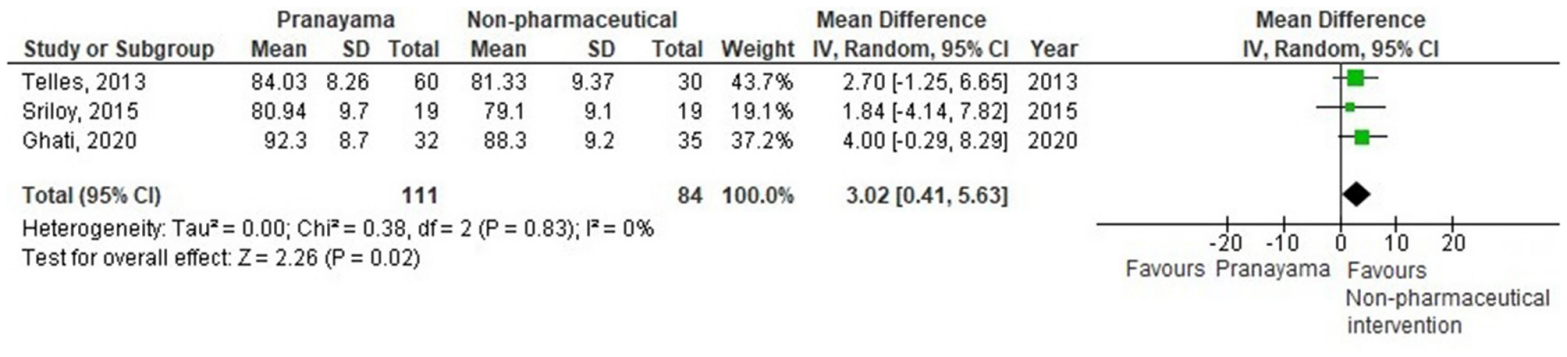
Pranayama vs.non-pharmaceutical intervention (DBP).

All subgroups favored yoga interventions in reducing DBP when compared to no intervention but not when compared to non-pharmaceutical intervention. Asana, pranayama, and dhyana and relaxation practice was more effective in reducing DBP compared to no intervention (−4.67 mmHg; −6.56– −2.77) but not compared to non-pharmaceutical interventions (−2.34 mmHg; −5.42–0.74) (Figures 8, 9). Pranayama was more effective in reducing DBP compared to no intervention (−5.38 mmHg; −8.03– −2.73) but not compared to non-pharmaceutical interventions (1.49 mmHg; −1.28–4.26) (Figures 10, 11). Interventions combining any two components of yoga could not be included in the meta-analysis as no two studies met the criteria to be pooled (46, 49, 58, 64, 67, 72).

### Details of the Yoga Interventions

#### Content

Of 20 effective interventions, 13 interventions incorporated 3 components of yoga: asana, pranayama, and dhyana and relaxation practices, 3 interventions combined pranayama, and dhyana, and relaxation practices, and 4 interventions used only pranayama. Fourteen effective interventions compared yoga with no intervention/no additional intervention, 5 compared with non-pharmaceutical intervention, and 1 study compared pranayama with another pranayama (74).

The majority of the studies did not define the style of the yoga (e.g., Hatha, Iyengar yoga, etc.) used as an intervention. Only 1 out of 20 effective interventions reported that Hatha yoga was used (56). The content of the effective yoga interventions was quite heterogeneous and they included 41 different types of asana, 19 different types of pranayama, and 11 different types of dhyana and relaxation practices. The most common practices were Bhujangasana (Cobra pose) (in 11 interventions), Anuloma-Viloma/Nadi Shodhana (Alternate nostril breathing) (in 14 interventions), and Shavasana (Corpse pose/deep relaxation) (in 9 interventions). The Sanskrit and English names of the yogic practices used in effective interventions and the number of RCTs using these practices are detailed in Supplementary Table 4.

The studies had a varying emphasis on each component. Four out of 13 effective interventions that incorporated 3 components of yoga did not report how much time was allocated to each component. Where reported, there was a balance in how much time allocated to each component on effective interventions, whereas ineffective studies were more focused on the asana and the duration of the asana practice was longer. The mean time allocated to asana was around 19 min, pranayama was around 9 min and dhyana and relaxation practice was around 14 min in effective studies. The mean time allocated to asana was around 42 min, pranayama was around 8 min and dhyana and relaxation practice was around 9 min in ineffective studies.

#### Structure

The duration of the effective yoga interventions ranged from 1 day (acute effect) to 12 weeks. Only 3 out of 20 effective studies evaluated the acute effects of yoga intervention. The frequency of sessions ranged from once a week to 7 days a week: 7 days a week (*n* = 5), 6 days a week (*n* = 4), 5 days a week (*n* = 3), and 3 days a week (*n* = 4). The duration of yoga sessions ranged from 10 min to 90 min and the mean duration of the yoga sessions was around 42 min. The most common session duration, frequency, and intervention duration of effective interventions was 45 min/session (in 5 interventions), 7 days/week (in 5 interventions), and 12 weeks (in 11 interventions).

Variations in the session duration and frequency of sessions resulted in an average weekly dose of 216 min in long-term effective interventions and the weekly dose ranged from 90 min to 420 min. The mean total dose of yoga interventions, considering the session length, session frequency, and total duration of yoga interventions, was around 2329 min (~39 h) and it ranged from around 560 min (~9 h) to 5,040 min (84 h).

There were differences between the effective and ineffective interventions in terms of the frequency, the weekly and total dose of the yoga interventions. Whereas, the most common session frequency was 7 days a week in effective interventions, the most common session frequency was 2 days a week (*n* = 7) in ineffective interventions. The mean weekly and total dose of effective yoga interventions was around 216 min and 2,329 min, respectively, whereas it was around 172 min and 2,071 min in long-term ineffective studies, respectively. The differences between the effective and effective interventions suggest that effective yoga interventions involved regular practice and tended to have higher weekly and total dose of yoga interventions.

#### Delivery Characteristics (Context, Supervision, and Intervention Uptake and Adherence)

Fifteen effective interventions were center-based, including a research/community center (*n* = 7), a clinic/hospital (*n* = 5) and academic institution (*n* = 2). Five interventions did not provide details of the centers where yoga interventions were practiced. Eight interventions were both center- and home-based and participants were recommended or required to practice at home.

Sixteen effective studies mentioned supervision of the yoga sessions but four studies did not provide any information about the yoga sessions' supervision status. Interventions were supervised by instructors with training on yoga (*n* = 9) and instructors without any training on yoga (*n* = 5). Of the studies that mentioned supervision of the yoga sessions, 2 studies did not provide further details about the person who supervised the sessions.

Four effective interventions did not report anything about the strategies used to increase intervention uptake and adherence and how compliant participants were to the intervention. A variety of strategies was used to increase attendance and adherence of participants to the intervention. Keeping attendance register (46, 48, 50, 55, 56, 65, 69, 75, 77), providing an audio recording, DVD, and/or training manuals for home practice (45, 46, 56, 63, 71, 77), recording home practice (45, 46, 63, 71), contacting the clinic/hospital to follow-up advice (45, 71, 77) and providing sessions at different times (56) were among the strategies used. Six out of 16 effective long-term studies reported the commitments of the participants to the intervention, and it ranged from 70 to 100% (48, 50, 56, 65, 69, 71).

Effective and ineffective interventions were similar in terms of the context, supervision, and strategies used to increase intervention uptake and adherence. However, participants' adherence to the intervention protocol tended to be higher in effective yoga interventions, where reported.

## Discussion

This systematic review synthesized the content, structure, and delivery characteristics of effective yoga interventions used for managing hypertension and compared these between effective and ineffective interventions. Eighteen, 14, and 20 interventions were effective in improving SBP, DBP, or either, respectively. Thirteen out of 20 effective interventions incorporated all the 3 major components of yoga, namely, asana, pranayama, and dhyana and relaxation practices and allocated similar durations to each component. These interventions involved regular practice and higher commitment of participants to the intervention protocol. The most common duration and frequency of effective yoga interventions were 45 min/session (in 5 interventions), 7 days/week (in 5 interventions), and 12 weeks (in 11 interventions). Fifteen were center-based, 8 were both center- and home-based, and 16 were supervised.

Yoga has been promoted as an aerobic form of exercise (8), and many of the studies had an emphasis on asana, however, this systematic review suggests that to be beneficial for hypertension yoga should include a balance of the three major components. One of the previous systematic reviews also showed that yoga interventions were more effective when they incorporated the three major components of yoga (10) but it is not possible to make a direct comparison as this review did not consider the time allocated to each component. A recent hypertension guideline suggests that moderate-intensity exercise should be practiced 5–7 days a week and stress reduction strategies like meditation should be introduced to the daily routine (8). This supports our finding that effective yoga interventions included regular practice. Another systematic review also found that DBP reductions were larger when yoga was practiced more than 3 sessions per week (34).

Adherence of the participants to the interventions is one of the most important points that affect the effectiveness of an intervention. To achieve high adherence, it is suggested that complex interventions, e.g., yoga, should combine counseling, self-monitoring, reinforcements, and supervision (8). We synthesized the methods used to increase intervention uptake and adherence in effective interventions. Although many of the effective studies included in this review kept attendance register and self-report of home-practice, they generally did not report the adherence rate. The guidance provided by the yoga instructors, and characteristics of the yoga instructors such as their training on the intervention protocol are critical for the effectiveness of the intervention and to ensure intervention fidelity and safe practice (78, 79). Many of the effective interventions included in this review reported if the intervention was supervised and if the instructors were trained in yoga but they mostly did not report if the instructors were trained in the intervention protocol. None of the previous systematic reviews synthesized the strategies used to increase intervention uptake and adherence, adherence rate, and yoga instructor characteristics.

### Methodological Quality of the Studies

Inadequate reporting was found in the included studies in terms of yoga intervention details, study design, and adverse events. Some important aspects of yoga interventions such as the characteristics of yoga providers, the existence of home practice, and attendance and adherence of participants to yoga sessions and home practice were not reported in 32, 59, and 48% of the included studies, respectively. In addition, the intervention development method and the details of the settings where yoga interventions were delivered were not adequately reported. The majority of the studies had low methodological quality with small sample size and short follow-up. The low methodological quality was due to either inadequate reporting or issues in the design of the studies.

As there was no sham or blinded comparison group in the majority of RCTs, the chances of performance bias (of participants) were high. It should be noted that yoga in the west is often considered as a standalone physical activity type of practice, however, traditionally and in many South Asian nations yoga incorporates a healthy lifestyle such as healthy diet, physical activity, smoking cessation, and restriction on alcohol intake (14, 80). Thus, yoga can be considered as a complex intervention, and participants in the yoga study arm might have been motivated to pursue a healthy lifestyle which is conducive to lowering blood pressure.

Future studies should ensure rigorous methodology, i.e., a larger sample size, longer follow-up, and ITT analysis, and reporting should be improved (79). In addition, although blinding of intervention deliverers is not possible in yoga interventions, blinding of participants can be achieved through sham therapies. Adverse events were also not explicitly reported in most of the studies, which are important to indicate the safety of yoga interventions. These issues should be addressed and reporting should be improved in future RCTs.

### Recommendations for Future Research and Practice

Despite the low quality and heterogeneity of included studies, our findings suggest yoga interventions may effectively manage hypertension, and provide an indication of the content, structure, and delivery characteristics, which may be most beneficial for managing hypertension. However, most of the included studies have not reported the intervention development process and it is hard to know whether these interventions were carefully thought out (e.g., their safety and acceptability) and comprehensive in their development. In addition, some intervention details were inadequately described (e.g., adherence and home practice), and it is difficult to replicate successful interventions. Thus, future studies should aim to systematically develop a yoga program for hypertension management in consultation with experts (e.g., yoga, hypertension) by using the synthesized findings of the effective interventions in this review. Then, they should aim to evaluate the intervention and implement it, if found effective. In addition, our review, showing that there are some narrative differences between effective and ineffective interventions, paved the way for future research and the next potential step can be to do a meta-analysis, using the methods developed for evaluating effects of individual components in complex interventions (81), to predict what intervention variables are associated with reductions in blood pressure.

### Strengths and Weaknesses

To the best of our knowledge, this is the first systematic review to synthesize the content, structure, and delivery characteristics of effective yoga interventions used for managing hypertension and to compare these between effective and ineffective interventions. This systematic review was conducted following a robust systematic process and using the JBI and the PRISMA guidelines by trained JBI accredited systematic reviewers. Everything was done by two reviewers independently with involvement of a third reviewer in case of any disagreements. Sixteen databases were searched with no date or language restrictions. Secondary hypertension and gestational hypertension were outside the scope of this review.

We tended to concentrate on the characteristics of effective yoga interventions and the effectiveness of the interventions was decided based on the statistical significance. This has a potential limitation due to the heterogeneity of the yoga interventions in terms of content, structure, and delivery characteristics and the inclusion of a lot of small studies in this review, which did not have enough power to achieve statistical significance. However, doing a meta-analysis was the best possible way to decide effectiveness because although studies had a comparator group, many of them compared pre-and post-test data within the group, and did not compare between intervention and control groups.

## Conclusion

Despite the low quality and heterogeneity of included studies, our findings suggest yoga interventions may effectively manage hypertension. The differences between the effective and ineffective interventions suggest that effective yoga interventions mostly incorporated asana, pranayama, and dhyana and relaxation practices and they had a balance between these three components and included regular practice. They were mostly delivered in a center and under supervision. Future studies should consider developing an intervention using the synthesized findings of this review before evaluating the effectiveness of yoga in managing hypertension.

## Data Availability Statement

The original contributions presented in the study are included in the article/Supplementary Material, further inquiries can be directed to the corresponding author.

## Author Contributions

GN conceptualized, designed, and conducted the study with the help of other authors, wrote the first draft of the manuscript and other authors contributed significantly to the revision of the manuscript. All authors read and approved the final manuscript.

## Funding

GN is a PhD student, funded by the Ministry of National Education of Turkey. This systematic review is a part of her PhD project.

## Conflict of Interest

The authors declare that the research was conducted in the absence of any commercial or financial relationships that could be construed as a potential conflict of interest.

## Publisher's Note

All claims expressed in this article are solely those of the authors and do not necessarily represent those of their affiliated organizations, or those of the publisher, the editors and the reviewers. Any product that may be evaluated in this article, or claim that may be made by its manufacturer, is not guaranteed or endorsed by the publisher.
