# Supplementary material for: Content, Structure, and Delivery Characteristics of Yoga Interventions for Managing Hypertension: A Systematic Review and Meta-Analysis of Randomized Controlled Trials
Source: Front Public Health. 2022 Mar 28;10:846231. doi: 10.3389/fpubh.2022.846231 (PMC8995771; doi:10.3389/fpubh.2022.846231)
Supplement: Supplementary file 1 [file Data_Sheet_1.pdf]

Supplementary Material

Search Strategy

Medline (Ovid) (1946-present)

1. exp Yoga/
2. exp Mind-Body Therapies/
3. exp Meditation/
4. yoga*.mp.
5. mind body therap*.mp.
6. yogi*.mp.
7. asana*.mp.
8. pranayam*.mp.
9. dhyan*.mp.
10. meditat*.mp.
11. ashtanga.mp.
12. bikram.mp.
13. hatha.mp.
14. iyengar.mp.
15. kripalu.mp.
16. kundalini.mp.
17. vinyasa.mp.
18. raja.mp.
19. radja.mp.
20. bhakti.mp.
21. jnana.mp.
22. kriya*.mp.
23. karma.mp.
24. yama.mp.
25. niyama.mp.
26. pratyahara.mp.
27. dharana.mp.
28. samadhi.mp.
29. bandha.mp.
30. mudra*.mp.
31. chanda.mp.
32. sivananda.mp.
33. or/1-32
34. exp Blood Pressure/
35. exp Essential Hypertension/
36. exp Hypertension/
37. (blood pressure or BP).mp.
38. hypertens*.mp.
39. pre?hypertens*.mp.
40. ((high or increase* or elevat*) adj2 (blood pressur* or BP)).mp.
41. ((diastolic or systolic or arterial) adj2 (pressur* or BP)).mp.

42. or/34-41
43. randomized controlled trial.pt.
44. controlled clinical trial.pt.
45. clinical trial.pt.
46. exp clinical trials as topic/
47. exp placebos/
48. exp random allocation/
49. exp double-blind method/
50. exp single-blind method/
51. exp cross-over studies/
52. ((random$ or control$ or clinical$) adj3 (trial$ or stud$)).tw.
53. (random$ adj3 allocat$).tw.
54. placebo$.tw.
55. ((singl$ or doubl$ or trebl$ or tripl$) adj (blind$ or mask$)).tw.
56. (crossover$ or (cross adj over$)).tw.
57. or/43-56
58. 33 and 42 and 57

Embase (Ovid) (1974-present)

1. exp Yoga/
2. exp Meditation/
3. yoga*.mp.
4. mind body therap*.mp.
5. yogi*.mp.
6. asana*.mp.
7. pranayam*.mp.
8. dhyan*.mp.
9. meditat*.mp.
10. ashtanga.mp.
11. bikram.mp.
12. hatha.mp.
13. iyengar.mp.
14. kripalu.mp.
15. kundalini.mp.
16. vinyasa.mp.
17. raja.mp.
18. radja.mp.
19. bhakti.mp.
20. jnana.mp.
21. kriya*.mp.
22. karma.mp.
23. yama.mp.
24. niyama.mp.
25. pratyahara.mp.
26. dharana.mp.
27. samadhi.mp.
28. bandha.mp.

29. mudra*.mp.
30. chanda.mp.
31. sivananda.mp.
32. or/1-31
33. exp Blood Pressure/
34. exp Essential Hypertension/
35. exp Hypertension/
36. (blood pressure or BP).mp.
37. hypertens*.mp.
38. pre?hypertens*.mp.
39. ((high or increase* or elevat*) adj2 (blood pressur* or BP)).mp.
40. ((diastolic or systolic or arterial) adj2 (pressur* or BP)).mp.
41. or/33-40
42. exp clinical trial/
43. exp randomized controlled trial/
44. exp randomization/
45. exp single blind procedure/
46. exp double blind procedure/
47. exp crossover procedure/
48. exp placebo/
49. randomi?ed controlled trial$.tw.
50. rct.tw.
51. random allocation.tw.
52. randomly allocated.tw.
53. allocated randomly.tw.
54. (allocated adj2 random).tw.
55. single blind$.tw.
56. double blind$.tw.
57. ((treble or triple) adj blind$).tw.
58. placebo$.tw.
59. exp prospective study/
60. Or/42-59
61. Exp case study/
62. Case report.tw.
63. Exp abstract report/ or exp letter/
64. Or/61-63
65. 60 not 64
66. 32 and 41 and 65

CINAHL (EBSCOhost) (1937-present)

S1. (MH "Yoga+")
S2. (MH “Mind body techniques+”)
S3. (MH “Meditation”)
S4. TX yoga*
S5. TX “mind body therap*”
S6. TX yogi*
S7. TX asana*
S8. TX pranayam*
S9. TX dhyan*
S10. TX meditat*
S11. TX ashtanga
S12. TX bikram
S13. TX hatha
S14. TX iyengar
S15. TX kripalu
S16. TX kundalini
S17. TX vinyasa
S18. TX raja
S19. TX radja
S20. TX bhakti
S21. TX jnana
S22. TX kriya*
S23. TX karma
S24. TX yama
S25. TX niyama
S26. TX pratyahara
S27. TX dharana
S28. TX samadhi
S29. TX bandha
S30. TX mudra*
S31. TX chanda
S32. TX sivananda
S33. OR/S1-S32
S34. (MH "Blood Pressure+")
S35. (MH "Essential Hypertension")
S36. (MH "Hypertension+")
S37. TX “blood pressure” or BP
S38. TX hypertens*
S39. TX pre*hypertens*
S40. TX ((high or increase* or elevat*) N2 (blood pressur* or BP))
S41. TX ((diastolic or systolic or arterial) N2 (pressur* or BP))
S42. OR/S34-S41
S43. S33 AND S42

PsycINFO (Ovid) (1806-present)

1. exp Yoga/
2. exp Mind Body Therapy/
3. exp Meditation/
4. yoga*.mp.
5. mind body therap*.mp.
6. yogi*.mp.
7. asana*.mp.
8. pranayam*.mp.
9. dhyan*.mp.
10. meditat*.mp.
11. ashtanga.mp.
12. bikram.mp.
13. hatha.mp.
14. iyengar.mp.
15. kripalu.mp.
16. kundalini.mp.
17. vinyasa.mp.
18. raja.mp.
19. radja.mp.
20. bhakti.mp.
21. jnana.mp.
22. kriya*.mp.
23. karma.mp.
24. yama.mp.
25. niyama.mp.
26. pratyahara.mp.
27. dharana.mp.
28. samadhi.mp.
29. bandha.mp.
30. mudra*.mp.
31. chanda.mp.
32. sivananda.mp.
33. or/1-32
34. exp Blood Pressure/
35. exp Essential Hypertension/
36. exp Hypertension/
37. (blood pressure or BP).mp.
38. hypertens*.mp.
39. pre?hypertens*.mp.
40. ((high or increase* or elevat*) adj2 (blood pressur* or BP)).mp.
41. ((diastolic or systolic or arterial) adj2 (pressur* or BP)).mp.
42. or/34-41
43. 33 and 42

Allied and Complementary Medicine Database (AMED) (Ovid) (1985-present)

1. exp Yoga/
2. exp Mind body medicine/
3. exp Meditation/
4. yoga*.mp.
5. mind body therap*.mp.
6. yogi*.mp.
7. asana*.mp.
8. pranayam*.mp.
9. dhyan*.mp.
10. meditat*.mp.
11. ashtanga.mp.
12. bikram.mp.
13. hatha.mp.
14. iyengar.mp.
15. kripalu.mp.
16. kundalini.mp.
17. vinyasa.mp.
18. raja.mp.
19. radja.mp.
20. bhakti.mp.
21. jnana.mp.
22. kriya*.mp.
23. karma.mp.
24. yama.mp.
25. niyama.mp.
26. pratyahara.mp.
27. dharana.mp.
28. samadhi.mp.
29. bandha.mp.
30. mudra*.mp.
31. chanda.mp.
32. sivananda.mp.
33. or/1-32
34. exp Blood Pressure/
35. exp Hypertension/
36. (blood pressure or BP).mp.
37. hypertens*.mp.
38. pre?hypertens*.mp.
39. ((high or increase* or elevat*) adj2 (blood pressur* or BP)).mp.
40. ((diastolic or systolic or arterial) adj2 (pressur* or BP)).mp.
41. or/34-40
42. 33 and 41

Cochrane Central Register of Controlled Trials (CENTRAL) (1996-present)

#1 MeSH descriptor: [Yoga] explode all trees
#2 MeSH descriptor: [Mind-Body Therapies] explode all trees
#3 MeSH descriptor: [Meditation] explode all trees
#4 Yoga* (Word variations have been searched)
#5 “mind body therapies” (Word variations have been searched)
#6 yogi* (Word variations have been searched)
#7 asana* (Word variations have been searched)
#8 pranayam* (Word variations have been searched)
#9 dhyan*(Word variations have been searched)
#10 meditat*(Word variations have been searched)
#11 ashtanga (Word variations have been searched)
#12 bikram (Word variations have been searched)
#13 hatha (Word variations have been searched)
#14 iyengar (Word variations have been searched)
#15 kripalu (Word variations have been searched)
#16 kundalini (Word variations have been searched)
#17 vinyasa (Word variations have been searched)
#18 raja (Word variations have been searched)
#19 radja (Word variations have been searched)
#20 bhakti (Word variations have been searched)
#21 jnana (Word variations have been searched)
#22 kriya* (Word variations have been searched)
#23 karma (Word variations have been searched)
#24 yama (Word variations have been searched)
#25 niyama (Word variations have been searched)
#26 pratyahara (Word variations have been searched)
#27 dharana (Word variations have been searched)
#28 samadhi (Word variations have been searched)
#29 bandha (Word variations have been searched)
#30 mudra* (Word variations have been searched)
#31 chanda (Word variations have been searched)
#32 sivananda (Word variations have been searched)
#33 {OR #1-#32}
#34 MeSH descriptor: [Blood Pressure] explode all trees
#35 MeSH descriptor: [Essential Hypertension] explode all trees
#36 MeSH descriptor: [Hypertension] explode all trees
#37 (“blood pressure” or BP) (Word variations have been searched)
#38 hypertens* (Word variations have been searched)
#39 pre?hypertens* (Word variations have been searched)
#40 ((high or increase* or elevat*) adj2 (blood pressur* or BP))
#41 ((diastolic or systolic or arterial) adj2 (pressur* or BP))
#42 {OR #34-#41}
#43 #33 and #42

Web of Science (1900-present)

#1 All=(yoga* OR “mind body therap*” OR meditation OR yogi* OR asana* OR pranayam* OR dhyan* OR meditat* OR ashtanga OR bikram OR hatha OR iyengar OR kripalu OR kundalini OR vinyasa OR raja OR radja OR bhakti OR jnana OR kriya* OR karma OR yama OR niyama OR pratyahara OR dharana OR samadhi OR bandha OR mudra* OR chanda OR sivananda)
#2 ALL=(“blood pressure” OR BP OR hypertens* OR pre-hypertens*)
#3 TS=(((high or increase* or elevat*) near/2 (“blood pressur*” or BP)) OR ((diastolic or systolic or arterial) near/2 (pressur* or BP)))
#4 #2 OR #3
#5 ALL=(“randomized controlled trial” OR “controlled clinical trial” OR “clinical trial” OR “clinical trials” OR placebo$ OR “random allocation” OR “double-blind method” OR “single-blind method” OR “cross-over studies”)
#6 TS=(((random$ or control$ or clinical$) near/3 (trial$ or stud$)) OR (random$ near/3 allocat$) OR ((singl$ or doubl$ or trebl$ or tripl$) near (blind$ or mask$)) OR (crossover$ or (cross near over$)))
#7 #5 OR #6
#8 #1 AND #4 AND #7

Turning Research into Practice (TRIP) (1997-present)

(yoga* OR “mind body therapies” OR yogi* OR asana* OR pranayam* OR dhyan* OR meditation OR meditate OR ashtanga OR bikram OR hatha OR iyengar OR kripalu OR kundalini OR vinyasa OR raja OR radja OR bhakti OR jnana OR kriya* OR karma OR yama OR niyama OR pratyahara OR dharana OR samadhi OR bandha OR mudra* OR chanda OR sivananda) AND (“blood pressure” OR BP OR hypertension OR hypertensive OR pre-hypertension OR prehypertension OR pre-hypertensive OR prehypertensive)

AYUSH Research Portal

Selected medical system > Yoga & Naturopathy > selected category > Clinical Research and Preclinical Research and Fundamental Research

A Bibliography of Indian Medicine (ABIM)

1. Blood pressure
2. BP
3. Hypertension
4. Hypertensive
5. Prehypertension
6. Pre-hypertension
7. Prehypertensive
8. Pre-hypertensive

Digital Helpline for Ayurveda Research Articles (DHARA)

1. Blood pressure [title]
2. BP [title]
3. Hypertension [title]
4. Hypertensive [title]
5. Prehypertension [title]
6. Pre-hypertension [title]
7. Prehypertensive [title]
8. Pre-hypertensive [title]

CAM-QUEST

Selected therapy – Mind-body medicine –> selected disease pattern – Cardiovascular diseases –> selected disease – Hypertension – selected study design –> Randomized trial

Directory of Open Access Journals (DOAJ)

1. yoga* and hypertens*
2. yoga* and blood pressure
3. yoga* and BP
4. yoga* and prehypertens*
5. yoga* and pre-hypertension
6. yoga* and pre-hypertensive
7. yogi* and hypertens*
8. yogi* and blood pressure
9. yogi* and BP
10. yogi* and pre-hypertension
11. yogi* and pre-hypertensive
12. yogi* and prehypertension
13. yogi* and prehypertensive

OpenGrey

1. yoga*
2. yogi*

EthOS

1. yoga [any word]
2. yogi [any word]
3. yogic [any word]

ProQuest Dissertations and Theses

1. (ti(yoga* OR mind body therap* OR yogi* OR asana* OR pranayam* OR dhyan* OR meditat* OR ashtanga OR bikram OR hatha OR iyengar OR kripalu OR kundalini OR vinyasa OR raja OR radja OR bhakti OR jnana OR kriya* OR karma OR yama OR niyama OR pratyahara OR dharana OR samadhi OR bandha OR mudra* OR chanda OR sivananda)
2. ti(blood pressure OR BP OR hypertens* OR pre?hypertens* OR ((high OR increase* OR elevat*) NEAR/2 ("blood pressur*" OR BP)) OR ((diastolic OR systolic or arterial) NEAR/2 (pressur* OR BP)))
3. 1 and 2
4. ab(yoga* OR mind body therap* OR yogi* OR asana* OR pranayam* OR dhyan* OR meditat* OR ashtanga OR bikram OR hatha OR iyengar OR kripalu OR kundalini OR vinyasa OR raja OR radja OR bhakti OR jnana OR kriya* OR karma OR yama OR niyama OR pratyahara OR dharana OR samadhi OR bandha OR mudra* OR chanda OR sivananda)
5. ab(blood pressure OR BP OR hypertens* OR pre?hypertens* OR ((high OR increase* OR elevat*) NEAR/2 ("blood pressur*" OR BP)) OR ((diastolic OR systolic OR arterial) NEAR/2 (pressur* OR BP)))
6. 4 and 5
7. 3 or 6

Excluded Studies with Reasons for Exclusion

Full-text studies excluded (n=47)

Related to population e.g., different target population (n=1)

Cade WT, Reeds DN, Mondy KE, Overton ET, Grassino J, Tucker S, et al. Yoga lifestyle intervention reduces blood pressure in HIV-infected adults with cardiovascular disease risk factors. *HIV Med* (2010) 11(6):379-88. doi: 10.1111/j.1468-1293.2009.00801.x.

Related to intervention e.g., not the main/only intervention, no adequate description of intervention (n=18)

Aivazyan TA, Zaitsev VP, Salenko BB, Yurenev AP, Patrusheva IF. Efficacy of relaxation techniques in hypertensive patients. *Health Psychol* (1988) 7:193-200.
Anderson DE, McNeely JD, Windham BG. Regular slow-breathing exercise effects on blood pressure and breathing patterns at rest. *J Hum Hypertens* (2010) 24(12):807-13. doi: 10.1038/jhh.2010.18.
Bell TP. Meditative practice cultivates mindfulness and reduces anxiety, depression, blood pressure, and heart rate in a diverse sample. *J Cogn Psychother* (2015) 29(4):343-55.
Bhasin MK, Denninger JW, Huffman JC, Joseph MG, Niles H, Chad-Friedman E, et al. Specific transcriptome changes associated with blood pressure reduction in hypertensive patients after relaxation response training. *J Altern Complement Med* (2018) 24(5):486-504. doi: 10.1089/acm.2017.0053.
Blom K, Baker B, How M, Dai M, Irvine J, Abbey S, et al. Hypertension analysis of stress reduction using mindfulness meditation and yoga: results from the HARMONY randomized controlled trial. *Am J Hypertens* (2014) 27(1):122-9. doi: 10.1093/ajh/hpt134.
Cort DA. A comparison of compliance to group meditation, individual meditation and didactic group training in a program to help lower blood pressure in Black adults. USA: The Florida State University (1988).
Dusek JA, Hibberd PL, Buczynski B, Chang BH, Dusek KC, Johnston JM, et al. Stress management versus lifestyle modification on systolic hypertension and medication elimination: a randomized trial. *J Altern Complement Med* (2008) 14(2):129-38. doi: 10.1089/acm.2007.0623.
Kaushik RM, Kaushik R, Mahajan SK, Rajesh V. Effects of mental relaxation and slow breathing in essential hypertension. *Complement Ther Med* (2006) 14(2):120-6. doi: 10.1016/j.ctim.2005.11.007.
Latha, Kaliappan K. Yoga, pranayama, thermal biofeedback techniques in the management of stress and high blood pressure. *J Indian Psychol* (1991) 9(1):36-46.
Dobos G. Comprehensive lifestyle modification for patients with hypertension and metabolic syndrome: a multicenter randomized controlled trial. [Unpublished]. Available from: https://www.medrxiv.org/content/medrxiv/suppl/2020/02/25/2020.02.23.20027029.DC1/2020.02.23.20027029-1.pdf
Nejati S, Zahiroddin A, Afrookhteh G, Rahmani S, Hoveida S. Effect of group mindfulness-based stress-reduction program and conscious yoga on lifestyle, coping strategies, and systolic and diastolic blood pressures in patients with hypertension. *J Tehran Heart Cent* (2015) 10(3):140-8.
Schroer S, Mayer-Berger W, Pieper C. Effect of telerehabilitation on long-term adherence to yoga as an antihypertensive lifestyle intervention: results of a randomized controlled trial. *Complement Ther Clin Pract* (2019) 35:148-53. doi: 10.1016/j.ctcp.2019.02.001.
Roche L, Hesse B. Application of an integrative yoga therapy programme in cases of essential arterial hypertension in public healthcare. *Complement Ther Clin Pract* (2014) 20(4):285-90.
Ublosakka-Jones C, Tongdee P, Pachirat O, Jones DA. Slow loaded breathing training improves blood pressure, lung capacity and arm exercise endurance for older people with treated and stable isolated systolic hypertension. *Exp Gerontol* (2018) 108:48-53. doi: 10.1016/j.exger.2018.03.023.
Ubolsakka-Jones C, Tongdee P, Jones DA. The effects of slow loaded breathing training on exercise blood pressure in isolated systolic hypertension. *Physiother Res Int* (2019) 24(4):e1785. doi: 10.1002/pri.1785.
van Montfrans GA, Karemaker JM, Wieling W, Dunning AJ. Relaxation therapy and continuous ambulatory blood pressure in mild hypertension: a controlled study. *BMJ* (1990) 300(6736):1368-72.

Ziv A, Vogel O, Keret D, Pintov S, Bodenstein E, Wolkomir K, et al. Comprehensive approach to lower blood pressure (CALM-BP): a randomized controlled trial of a multifactorial lifestyle intervention. *J Hum Hypertens* (2013) 27(10):594-600. doi: 10.1038/jhh.2013.29.
Srinivasan B, Rajkumar D. Effects of slow breathing on blood pressure and end tidal carbon dioxide in hypertension: randomised controlled trial. *J Clin Diagn Res* (2019) 13(9):YC01-YC3. doi: 10.7860/JCDR/2019/42327.13121.

Related to comparator (n=1)

Kapoor G. An analytical study to find out the effects of four asanas on decreasing blood pressure and to compare immediate effects on blood pressure of four different sequences of common asanas used in treatment of hypertension. *Indian J Phys Ther* 2(1):46-50.

Related to study design e.g., not an/unclear RCT (n=21)

Agte VV, Jahagirdar MU, Tarwadi KV. The effects of Sudarshan Kriya Yoga on some physiological and biochemical parameters in mild hypertensive patients. *Indian J Physiol Pharmacol* (2011) 55(2):183-7.
Andrews G, MacMahon SW, Austin A, Byrne DG. Hypertension: comparison of drug and non-drug treatments. *Br Med J (Clin Res Ed)* (1982) 284(6328):1523-6.
Bhavanani AB, Madanmohan, Sanjay Z. Immediate effect of chandra nadi pranayama (left unilateral forced nostril breathing) on cardiovascular parameters in hypertensive patients. *Int J Yoga* (2012) 5(2):108-11. doi: 10.4103/0973-6131.98221.
Bhavanani AB, Sanjay Z, Madanmohan. Immediate effect of sukha pranayama on cardiovascular variables in patients of hypertension. *Int J Yoga Therap* (2011) (21):73-6.
Damodaran A, Malathi A, Patil N, Shah N, Suryavansihi, Marathe S. Therapeutic potential of yoga practices in modifying cardiovascular risk profile in middle aged men and women. *J Assoc Physicians India* (2002) 50(5):633-40.
Das D, Manik R, Gartia R. Effects of integrated approach of yoga (IAY) on essential hypertension. *J Evid Based Med Healthc* (2015) 2:4925-38. doi: 10.18410/jebmh/2015/689.
Datey K, Deshmukh S, Dalvi C, Vinekar S. Shavasan a yogic exercise in the management of hypertension. *Angiologie* (1969) 20(6):325-33. doi: 10.1177/000331976902000602.
Dhameja K, Singh S, Mustafa MD, Singh KP, Banerjee BD, Agarwal M, et al. Therapeutic effect of yoga in patients with hypertension with reference to GST gene polymorphism. *J Altern Complement Med* (2013) 19(3):243-9. doi: 10.1089/acm.2011.0908.
Gowtham L, Vasanthi B, Jayshree N, Ambika, Halder N, Velpandian T. Effects of yoga in type 2 diabetes mellitus with hypertension: alteration in RBC morphology as a marker for oxidative stress. *Indian J Physiol Pharmacol* (2016) 62(1):51-8.
Miles SC, Chun-Chung C, Hsin-Fu L, Hunter SD, Dhindsa M, Nualnim N, et al. Arterial blood pressure and cardiovascular responses to yoga practice. *Altern Ther Health Med* (2013) 19(1):38-45.
Mizuno J, Monteiro HL. An assessment of a sequence of yoga exercises to patients with arterial hypertension. *J Bodywork Mov Ther* (2013) 17(1):35-41. doi: 10.1016/j.jbmt.2012.10.007.
Murthy SN, Rao NS, Nandkumar B, Kadam A. Role of naturopathy and yoga treatment in the management of hypertension. *Complement Ther Clin Pract* (2011) 17(1):9-12. doi: 10.1016/j.ctcp.2010.08.005.
Ranawat R, Agarwal V, Sharma V, Sharma P. Prevention and management of hypertension and associated disorders by Bhastrika pranayama (A breathing exercise). *JAHM* (2015) 3(3):7-10.
Santaella DF, Araujo EA, Ortega KC, Tinucci T, Mion D, Jr., Negrao CE, et al. Aftereffects of exercise and relaxation on blood pressure. *Clin J Sport Med* (2006) 16(4):341-7. doi: 10.1097/00042752-200607000-00010.
Selvamurthy W, Sridharan K, Ray US, Tiwary RS, Hegde KS, Radhakrishan U, et al. A new physiological approach to control essential hypertension. *Indian J Physiol Pharmacol* (1998) 42(2):205-13.
Shaha R. Effect of Rajyoga meditation on psychological and physical well-being among hypertensive, diabetic and coronary artery disease patients [Ph.D.]. Ann Arbor: Devi Ahilya Vishwavidyalaya (2013).
Shrikrishna. Comprehensive approach for the treatment of essential hypertension through yoga. *Yoga-Mimamsa* (1990) 29(1):67-70.

Vasantha Priya J, Kanniammal C, Mahendra J, Valli G. Impact of yoga on blood pressure and quality of life in patients with hypertension. *Int J Pharm Clin Res* (2017) 9(5):413-16.
Walsh KL. Yoga as a complementary therapy in the management of hypertension. USA: The University of North Carolina at Chapel Hill (2019).
Wolff M, Sundquist K, Larsson Lönn S, Midlöv P. Impact of yoga on blood pressure and quality of life in patients with hypertension - a controlled trial in primary care, matched for systolic blood pressure. *BMC Cardiovasc Disord* (2013) 13(1):111. doi: 10.1186/1471-2261-13-111.
Deepa T, Sethu G, Thirrunavukkarasu N. Effect of yoga and meditation on mild to moderate essential hypertensives. *J Clin Diagn Res* (2012) 6(1):21-6.

Combination of the above four criteria (n=7)

Metri KG, Pradhan B, Singh A, Nagendra HR. Effect of 1-week yoga-based residential program on cardiovascular variables of hypertensive patients: a comparative study. *Int J Yoga* (2018) 11(2):170-4. doi: 10.4103/ijoy.IJOY_77_16.
Packyanathan J, Preetha S. Comparison of the effect of yoga, zumba and aerobics in controlling blood pressure in the Indian population. *J Family Med Prim Care* (2020) 9(2):547-51. doi: 10.4103/jfmpc.jfmpc_607_19.
Patel C. Yoga and biofeedback in the management of hypertension. *J Psychosom Res* (1975) 19(5-6):355-60. doi: 10.1016/0022-3999(75)90014-8.
Telles S, Sharma SK, Balkrishna A. Blood pressure and heart rate variability during yoga-based alternate nostril breathing practice and breath awareness. *Med Sci Monit Basic Res* (2014) 20:184-93. doi: 10.12659/MSMBR.892063.
Pramanik T, Sharma HO, Mishra S, Mishra A, Prajapati R, Singh S. Immediate effect of slow pace Bhastrika pranayama on blood pressure and heart rate. *J Altern Complement Med* (2009) 15(3):293-5. doi: 10.1089/acm.2008.0440.
Patel C. 12-month follow-up of yoga and bio-feedback in the management of hypertension. *Lancet* (1975) 1(7898):62-4. doi: 10.1016/s0140-6736(75)91070-3.
Yadav RK, Ray RB, Vempati R, Bijlani RL. Effect of a comprehensive yoga-based lifestyle modification program on lipid peroxidation. *Indian J Physiol Pharmacol* (2005) 49(3):358-62.

Ongoing Studies (n=8)

Effect of yoga Nidra on blood pressure in patients with resistant hypertension: a randomized controlled trial. India. CTRI/2020/02/023365
Effect of OM chanting on emotional regulation and its effect on hypertension. India. CTRI/2020/02/023458
Effect of yoga nidra with Om chanting on the control of blood pressure and hypertension. India. CTRI/2020/02/023400
Comparison of the effectiveness of interventions based on the Eastern mind-body approach on cardiovascular responses and stress reduction in prehypertensive subjects. Iran. IRCT20200607047679N1
Immediate effect of ice massage to head and spine and cooling pranayamas on heart rate variability in hypertensive individuals a comparitive study. India. CTRI/2019/12/022378
Immediate effect of Nadishodhana pranayama and bhramari pranayama on heart rate variability in hypertensive individuals a comparative study. India. CTRI/2019/12/022263
Efficacy of integrated yoga as a comprehensive intervention among paramilitary personnel with hypertension – YCT. India. CTRI/2020/06/026172
Effects of yoga training on cardiovascular reactivity to psychological stress in patients with hypertension. Taiwan. NCT03274193

Table S1 Study characteristics of included RCTs

Author and year	Country	Population characteristics	Sample size	Intervention	Comparator	SBP (mmHg)	DBP (mmHg)	Adverse events	Final follow-up (in weeks)	Loss to follow-up	SBP and DBP data extraction time-point (in weeks)
						Baseline	Baseline				
Murugesan, 2000	India	Both sexes, HTN	33 (I1=11, C1=11, C2=11)	I=Yoga	C1=No intervention C2=Antihypertensive treatment	I=156.45±9.21 C1=155.45±10.34 C2=158.63±11.52	I=108.63±9.92 C1=109.09±9.60 C2=106.45±10.32	NR	11	NR	11
McCaffrey, 2005	Thailand	Age (mean): 56.45, both sexes (F:35), essential HTN, not on medication	61 (I= 32, C=29)	I=Yoga	C=Usual care	I=160.89±10.37 C=160±13.35	I=98.52±8.33 C=98.30±7.10	NR	8	No time/inability to get to the yoga centers: I=5 Started taking antihypertensive drugs: C=1 Moved out of the province: C=1	8
Kettner, 2009	Germany	Age (mean): 51.50, only male, HTN, on medication	340 (I=173, C=167)	I=Viniyoga	C=Progressive muscle relaxation	I=131.11±18.19 C=132.76±18.12	I=81.39±10.75 C=80.67±10.89	NR	24	Discharge from the clinic: I=27, C=9	3
Mourya, 2009	India	Both sexes (F:29), stage I essential HTN, on medication or not, yoga-naïve	60 (I1=20, I2=20, C=20)	I1=Slow yogic breathing I2=Fast yogic breathing	C=No intervention	I1=145.90±9.21 I2=146.80±9.25 C=146.93±8.67	I1=91.95+9.12 I2=91.09+8.13 C=91.01±8.49	NR	12	Did not practice regularly: 2 Could not learn the technique: 3 Started dieting:2	12
Saptharishi, 2009	India	Age (mean): 22.50, both sexes (F:34), prehypertension and HTN	113 (I=30, C=30, C2=30, C3=30)	I=Yoga	C=No intervention C2=Salt intake reduction C3=Physical exercise	I=127.60± 9.90 C1=123.80±10.80 C2=123.10±8.10 C3=128.40±7.60	I=85.80±6.70 C1=83.20±7.20 C2=83.70±6.80 C3=87.40±4.80	NR	8	Did not give consent: I1=3, C2=2, C3=2 Drop-out: I= 6, C1=1, C2=3, C3=1	8
Khadka, 2010	Nepal	Age (mean): 43.53, both sexes, essential HTN, on medication and salt reduction, yoga-naïve	14 (I=7, C=7)	I=Yoga	C=No intervention	I=143±14.07 C=140±19.25	I=98±5.18 C=86±7.40	NR	6	NR	6
Cohen, 2011	USA	Age (mean): 48.24 both sexes (F:39), prehypertension	78 (I=46, C=32)	I=Iyengar yoga	C=Enhanced usual care	I=132±15.29 C=135±16.70	I=83±10.19 C=82±11.13	NS (n=3)	12	Protocol criteria: I=4	6

		and stage 1 HTN, not on medication yoga-naïve								Personal decision: I=7	
Bhavanani, 2012	India	Age (mean): 56.66, both sexes (F:14), essential HTN, on medication, T2DM, attending regular therapy sessions at ACYTER	29 (I=15, C=14)	I=Pranava pranayama	C=Sham relaxation	I=134.30±3.80 C=136.30±4.10	I=77.20±1.30 C=77.40±2.20	NR	Acute effect	None	-
Shantakumari, 2012	India	Age (mean): 44.98, both sexes (F:48), HTN, T2DM	100 (I=50, C=50)	I=Yoga+ Antidiabetic treatment	C=Only antidiabetic treatment (Sulfonylureas)	I=141.71±9.80 C=137.37±28.77	I=90.57±4.07 C=88.23±17.05	NR	12	None	12
Telles, 2013	India	Age (mean): 49.70, both sexes (F:30), uncomplicated essential HTN, on medication, familiar with the yoga for at least 6 months	90 (I1=30, I2=30, C=30)	I1=Anuloma-Viloma/ Nadi Shodhana (Alternate nostril breathing) I2=Breath awareness	C=Reading a magazine	I1=133.67±14.26 I2=130.67±16.39 C=140.83±14.22	I1=85.67±8.58 I2=84.33±10.73 C=81.20±9.42	None	Acute effect	None	-
Hagins, 2014	USA	Age (mean): 54.56, both sexes (F:58), prehypertension and stage I HTN	84 (I=45, C=39)	I=Ashtanga Yoga	C=Non-aerobic exercise	I=135.53±9.79 C=133.80±9.86	I=80.82±7.33 C=80.17±7.49	None	12	Lost to follow-up: I=9, C=7	12
Patil, 2014a	India	Age (mean): 68.92, male-only, stage I HTN, not on medication	57 (I=28, C=29)	I=Yoga	C=Walking	I=146.07±5.18 C=145.72±5.90	I=74.25±4.68 C=75.52±5.21	NR	12	Did not appear for the post-data due to domestic reasons: I=2, C=1	12
Patil, 2014b	India	Age (mean): 69.47, male only, stage I HTN, not on medication	42 (I=21, C=21)	I=Yoga	C=Lifestyle modification (stretching exercise and brisk walk)	I=147.23±5.62 C=147±5.82	I=74.95±3.80 C=75.52±5.43	NR	6	NR	6
Sujatha, 2014	India	Both sexes (F:128), stage 1 and 2 HTN, on medication, yoga-naïve	238 (I= 118, C=120	I=Hatha Yoga	C=Wait-list control group receiving no treatment	I=152.75±11.57 C=152.85±10.68	I=94.51±6.92 C=94.77±6.40	NR	12	None	12
Patil, 2015	India	Age (mean): 68.90, male-only,	60 (I=30, C=30)	I=Yoga	C=Brisk walking	I=146.96±5.70 C=145.86±6.30	I=74.13±4.58 C=75.53±5.50	NR	12	None	12

		Isolated systolic HTN with increased PP=60 mmHg, not on medication, yoga-naïve									
Pushpanathan, 2015	India	Essential HTN	70 (I=34, C=36	I=Yoga + Antihypertensive treatment	C=Yoga+ Antihypertensive treatment	I=124.8±8.10<br>C=126.47±11.21	I=80.44±7.02<br>C=83.2±7.11	NR	12	Discontinued the intervention: I=4, Lost to follow-up: C=6	12
Prakash, 2015	India	Uncomplicated HTN, on medication	50 (I=25, C=25)	I=Yoga+ Antihypertensive	C=Only antihypertensive treatment	I=149±18.48<br>C=150±20.36	I=91±13.08<br>C=91±11.76	NR	8	NR	8
Sriloy, 2015	India	Age (mean): 48.84, both sexes (F:10), uncomplicated HTN	46 (I=23, C=23)	I=Yoga breathing	C= Acupuncture	I=139.50±17.50<br>C=143.70±19.70	I=81.15±11.30<br>C=80.50±10	NR	Acute effect	Refused to participate: I= 4, C=3<br>Did not turn for the post-data: C=1	-
Thiyagarajan, 2015	India	Age (mean):43.29, both sexes (F:38), prehypertensive, not on medication	192 (I=96, C=96)	I=Yoga + Lifestyle modification	C=Lifestyle modification	I=127±6<br>C=127±5	I=85±4<br>C=85±4	NR	12	Excluded based on biochemical results: I=4, C=4, Asymptomatic, lack of self-motivation, family commitments and no immediate health benefit: I=41, C=43	12
Cohen, 2016	USA	Age (mean): 49.60, both sexes (F: 70), prehypertension and stage I HTN, not on medication, yoga-naïve	137 (I=43, C1= 48, C2= 46)	I=Forrest yoga	C1=BPEP program (health education classes and a walking program)<br>C2=COMBO (Forrest yoga and BPEP)	I=132.60±16.10<br>C1=134.20±14.60<br>C2=132.80±15.20	I=80.60±13.60<br>C1=82.20±13.10<br>C2=81.10±13.20	None	24	Protocol criteria: I=8, C1=4, C2=7<br>Personal decision: I=5, C1=8, C2=6	12
Punita, 2016	India	Age (mean): 43.38, both sexes (F:11), HTN, on medication	80 (I=40, C=40)	I=Yoga+ antihypertensive drugs	C=Antihypertensive drugs	I=124.80±8.10<br>C=126.47±11.21	I=80.44±7.02<br>C=83.2 ± 7.11	NR	12	Continued to treatment in their native place: I=14, C=10<br>Attendance less than 60%: I=1	12

Wolff, 2016	Sweden	Age (mean): 64.70, both sexes (F:99), prehypertension and stage I HTN, on medication or not	191 (I=96, C=95)	I=Home-based Kundalini yoga	C=Usual care	I=148.80±11.60 C=150±10.60	I=88.30±6.10 C=88.10±5.70	None	12	No time/feeling stressed: I=4 No reason: I=4, C=2 Illness: I=3, C=5 Unable to attend follow-up: C=2	12
Roche, 2017	Spain	Age (mean): 57.69, both sexes, essential HTN, yoga-naïve	100 (I1= 25, I2=25, I3=25, C=25)	I1=Yoga I2=Pranayama I3=Himalayan Tradition Meditation	C=One time lecture on HTN and healthy lifestyle habits given after one month	_	_	NR	8	Did not consent: I1=3, I2=2, I3=4, C=6 Drop-out: I1=8, I2=4, I3= 9, C=9	8
Shetty, 2017	India	Both sexes, prehypertension and stage I HTN, on medication, yoga-naïve	60 (I=30, C=30)	I=Pranayama	C=Wait-list control group	I=148.20±7.90 C=153.60±9.50	NR	NR	4	None	4
Supriya, 2017	Hong Kong	Age (mean): 57.57, both sexes (F:65), prehypertension, MetS	97 (I= 52, C=45)	I=Yoga	C=No intervention (monthly contact to monitor their health status)	I=140.27±13.46 C=141.71±13.85	I=84.52±7.80 C=85.78±8.50	NR	52	NR	52
Misra, 2018	USA	Age (mean): 60.80, both sexes (F:48), uncontrolled HTN, on medication or not	83 (I1=23, I2=38, C=22)	I1=In-class instruction yogic breathing I2=DVD/YouTube instruction yogic breathing	C=Usual care+ recording dinner time	I1=155.40±15.90 I2=152.50±15.50 C=149.20±23.70	I1=87.63±11.33 I2=84.70±5.96 C=89.86±9.52	NR	10	Did not show up: I1=15, I2=7, C=8 Dropped out: I1=6, I2=12, C=2	10
Cramer, 2018	Germany	Age (mean): 58.70, both sexes (F:54), primary arterial HTN, on medication	75 (I1=25, I2=25, C=25)	I1=Yoga with postures I2= yoga without postures	C=Wait-list control group receiving usual care	I1=135.40±11.60 I2=131.70±11.30 C=133.30±9.80	I1=82.50± 10.30 I2=81.10± 10.90 C=81.10±9.10	No severe adverse events occurred*	28	Acute Illness: I1=3, I2=2 Brake off contact: I2=1	12
Ankolekar, 2019	India	Prehypertension	102 (I= 51, C=51)	I=Yoga	NR	I=134±4.96 C=133.45±4.76	I=85.95±2.54 C=86.03±5.03	NR	24	NR	12
Gadgil, 2019	India	Female only, prehypertension , postmenopause	60 (I=30, C=30)	I=Fast pranayama	C=Slow pranayama	I=115.67±1.34 C=119.33±9.04	I=74.53±4.16 C=78.20±6.15	NR	Acute effect	NR	-

Fetter, 2020	Brazil	Age (mean): 59.08, female only, HTN, on medication or not, post-menopause, yoga-naïve	50 (I=10, C1=9, C2=14)	I=Yoga+ ujjayi pranayama	C1=Stretching + ujjayi pranayama C2=Stretching or Yoga alone	I=137.40±12.01 C1=141.20±14.10 C2=142.90±21.70	I=86.23±6.89 C1=87.15±8.94 C2=82.66±10.10	NR	12	Lost to follow-up:5 Declined to participate: 12	12
Ghati, 2020	India	Age (mean): 48.89, both sexes (F:31), essential HTN, on medication, yoga-naïve	70 (I=35, C=35)	I=Bhramari pranayama	C=Placebo slow breathing exercise	I=131.75±9.67 C=127.80±12.91	I=91.37±7.72 C=88.05±9.81	NR	Acute effect	Missing data: I=3	-
Sathe, 2020	India	Age (mean): 60.14, both sexes (F:21), HTN, on medication	42 (I=21, C=21)	I=Bhramari pranayama	C=Buteyko breathing	I=139.61±6.04 C=140.75±21.27	I=81.28±5.22 C=83.15±10.75	NR	Acute effect	None	-
Thanalakshmi, 2020	India	Age (mean):38.5, both sexes (F:23), primary HTN, on medication, yoga-naïve	100 (I=50, C=50)	I=Sheetali pranayama	C= No additional intervention	I=143.88±10.60 C=146.10±11.28	I=87.12±7.40 C=88.90±9.88	None	12	I=10 (health issues: 6, participated in other activities:2, missed more than 2 weeks of yoga:2) C=8 (health issues: 7, participated in other activities:1)	12
Dhungana, 2021	Nepal	Age (mean): 47.74, both sexes (F: 58), stage I HTN, on medication or not, yoga-naïve	121 (I=61, C=60)	I=Yoga + health education	C=Health education	I= 141.70±9.10 C= 136.90±9.00	I= 90.30±5.40 C= 89.40± 5.10	None	12	No stated reasons: I=2, C=1	12

Only yoga-related interventions were mentioned under intervention, and no intervention or any other active interventions were mentioned under comparator. Blood pressure results were reported as mean and SD.

* The adverse events registered in the yoga group with asana were: headache (n=3), shoulder pain (n=1), knee pain (n=1), tendinitis (n=1), meniscus rupture (n=1), prolapsed intervertebral disc (n=1), hypertensive emergency (n=1) and mental problems (n=1) and in the yoga intervention without asana were tinnitus (n=1) and knee pain (n=1). The adverse events registered in control group were headache (n=1), neck pain (n=1) and sleep disturbances (n=1).

I: Intervention, C: Control, NR: Not reported, HTN: Hypertension, ACYTER: The Advanced Centre for Yoga Therapy Education and Research, JIPMER: Jawaharlal Institute of Postgraduate Medical Education & Research

Table S2 Intervention details of the included RCTs

Author/ Year	Intervention development	Yoga sessions: structure (duration and frequency)	Yoga sessions: delivery characteristics (context and instructor)	Yoga sessions: delivery characteristics (intervention uptake and adherence)	Asana	Pranayama	Dhyana and relaxation	Extra features
Murugesan, 2000	NR	I=30 min x 2/day x 6 days/ week x 11 weeks	NR	NR	Pawanamuktasana (Wind releasing pose), Ardha Halasana (Half plough pose), Viparita Karani (Legs up the wall), Ardha Matsyendrasana (Half lord of the fishes pose), Makarasana (Crocodile pose), Bhujangasana (Cobra pose), Ardha Salabhasana (Half locust pose), Vakrasana (Twisted pose), Vajrasana (Thunderbolt pose), Yoga Mudrasana (Psychic union/Yoga seal pose), Urdhva Dhanurasana/ Chakrasana (Wheel pose), Tadasana (Mountain pose)	Anuloma-Viloma/Nadi Shodhana (Alternate nostril breathing)	Om recitation and meditation, Shavasana (Corpse pose/ Deep relaxation)	_
McCaffrey, 2005	- Yoga for Health was modified by the researcher	I=63 min/day x 3 days/ week x 8 weeks	-In two temporary yoga training centres<br>-Supervised by a trained research assistant	- Booklets and a cassette tape of yoga guidence were given<br>- Self-reporting of home practice via a diary	Dhanurasana (Bow pose), Bhujangasana (Cobra pose), Makarasana (Crocodile pose), Matsyasana (Fish pose), Janu Sirsasana (Head-to-knee forward bend pose), joint exercise, Padmasana (Lotus pose), Tadasana (Mountain pose), Vajrasana (Thunderbolt pose), Urdhva Dhanurasana/ Chakrasana (Wheel pose), Yoga mudrasana (Psychic union/Yoga sea pose), Yoni mudra	Pranayama (NS)	Shavasana (Corpse pose/ Deep relaxation)	-Intervention included health information and group support in learning yogic principles and stress reduction techniques
Kettner, 2009	NR	I=45 min/ day x 5 days/week x3 weeks	-At a clinic<br>-Supervised by a yoga teacher	NR	8 sets: Tadasana (Mountain pose), Urdhva hastasana (Upward salute), Ardha Uttanasana (Half forward bend), 4 times both sides: Virabhadrasana I (Warrior I), 8 sets: Paschimottanasana (Seated forward bend), 4 times both sides: Vakrasana (Spinal twist), 8 sets: Bharmanasana (Table top pose), Balasana (Child pose), 8 sets: Setu Bandha Sarvangasana (Bridge pose), 8 sets: Apanasana (Knees to chest pose), 4 sets: Chair Urdhva Hastasana (Chair Upward salute)	8 sets: Bhramari pranayama ((Bumblee) Bee breathing)	NR	-Modified if needed without changing the standardised basic concept and chair was used to do some of the practices

Mourya, 2009	NR	I1=15min x 2/day x 7 days/week x 12 weeks<br><br>I2=15min x 2/day x 7 days/week x 12 weeks	-At research centre<br>-Supervised by a yogic instructor for the first few days until technique was learnt	-Cassettes were given<br>-Flexible practice time was offered<br>- They were advised to time breathing rate<br>-Accuracy of the practice was checked on visits to the department<br>-A questionnaire was given every month to ensure motivation and compliance	I1 and I2= Padmasana (Lotus pose): 5 min	I1= Anuloma-Viloma/Nadi Shodhana (Alternate nostril breathing):15 min (5–6 breaths per min)<br>I2= Bhastrika pranayama (Bellow breathing) (The procedure was repeated 4 to 5 times over a period of 15 min with 3 min of rest after one min of practice).	NA	I1 and I2=<br>-Pranayama was taught everyday for the first 2 weeks and then, themselves practised twice daily 10–12 hours apart<br>-Classes had 4–6 participants and held at 9am
Saptharishi, 2009	NR	I=30-45 min/day x 5 days/ week x 8 weeks	-Supervised by a qualified yoga teacher of the institute (JIPMER)	NR	Talasana (Palm tree pose), Utkatasana (Chair pose), Trikonasan (Triangle pose), Ardha-matsyendrasan (Half lord of the fishes pose), Bakasana (Crow pose), Pawanmuktasan (Wind releasing pose), Navasana/ Naukasan (Boat pose), Matsyasana (Fish pose), Pashchimottanasana (Seated forward bend), Halasana (Plough pose), Bhujangasana (Cobra pose), Salabhasana (Locust pose), Sarvangasana (Shoulder stand)	Mukha Bhastrika (Downward facing bellow breath), Mahat-yoga pranayam (Complete deep breathing), Anuloma-Viloma/Nadi Shodhana (Alternate nostril breathing), Savitri pranayam (Rhytmic breath)	Shavasana (Corpse pose/ Deep relaxation)	–
Khadka, 2010	NR	I= 30 min/day x 6 days/ weeks x 6 weeks	-At hospital<br>-Supervised by a trained yoga instructor	NR	5 min (40 sec each): Suksha Vyayama (Loosening practices): Manibandha, Shakt Vikasaka, Ardha Bhuja Shakti Vikasaka, Purna Shakti Vikasaka (Arms rotation), Anguli Shakti Vikasaka, Kamar Chakrasana, Vakshasthal Shakti Vikasaka and Uder Shakti Vikasaka, 6-7 min (40 sec each): Tarasana (Star pose), Trikonasana (Triangle pose), Gomukhasana (Cow face pose), Shashankasana (Rabbit pose), Padmasana (Lotus pose), Bhujangasana (Cobra pose), Hardhayastambhasana, Navasana/Naukasana (Boat pose), Makarasana (Crocodile pose)	Anuloma-Viloma/Nadi Shodhana (Alternate nostril breathing): 4 min	Shavasana (Corpse pose/ Deep relaxation): 5 min, Meditation (NS): 5 min.	–

Cohen, 2011	-Developed by IY-certified instructor, Joan White, with input from B.K.S. Iyengar	I= 70 min/day x 2 days/ week x 6 weeks +70 min/day x 1 day/week x 6 weeks	- Supervised by two IY-certified instructors<br>- Encouraged to practise at home	-DVD was given for home practice<br>-Self-reporting of home practice via a diary	Cross bolsters: 5min, Supta Baddha Konasana (Reclining bound angle pose): 5min, Supta Swastikasana (Supine auspicious pose): 5min/side, Bharadvajasana (Torso stretch pose): 3 × 30 s/side, Pawanmuktasana (Wind releasing pose): 5 min, Adho Mukha Virasana (Downward facing hero pose): 5min, Adho Mukha Swastikasana (Downward facing auspicious pose):1min/side, Adho Mukha Svanasana (Downward facing dog): 1min, Uttanasana (Standing forward bend): 1min, Janu Sirsasana (Head to knee forward bend): 1min/side, Upavistha Konasana (Wide-angle seated forward bend): 3min, Paschimottanasana (Seated forward bend) 1min	Ujjayi pranayama (the conqueror): 5min	Shavasana (Corpse pose/ Deep relaxation): 10 min (5 min at the beginning and 5 min at the end)	-Classes were closed to the public<br>-Classes had 2–10 participants<br>-Props were used if needed
Bhavanani, 2012	NR	I= 10 min	-In the ACYTER, JIPMER	NA	NA	6 min in total: Pranav pranayama (Om meditation breathing in corpse pose): 3 rounds, Dirga/Mahat pranayama (Full yogic breath/ three-part breath):3 rounds	Shavasana (Corpse pose/ Deep relaxation): 4 min (2 min at the beginning and 2 min at the end)	_
Shantakumari, 2012	-Based on literature review done by yoga specialists	I=60 min/ day x 7 days/ week x 12 weeks	- In the holistic medicine clinic<br>-Supervised by an experienced yoga teacher<br>-Encouraged to practise at home	-Personalised yoga program for home practice was given<br>- Self-reporting of home practice<br>-Contacted study centre every month for follow-up advice	Surya Namaskara (Sun salutation): 5 min, Yoga Mudrasana (Psychic union/ Yoga seal pose): 2 min, Vajrasana (Thunderbolt pose): 2 min, Vakrasana (Twisted pose): 2 min, Paschimottasana (Seated forward bend): 2 min, Pawanamuktasana (Wind releasing pose): 2 min, Sashankasana (Rabbit pose): 2 min, Ustrasana (Camel pose):<br>2 min, Bhujangasana (Cobra pose): 2 min, Dhanurasana (Bow pose): 2 min, Ardhakati Chakrasana (Lateral arc pose): [illegible] min, Parivrtta Trikonasana (Revolved triangle pose): 2 min	5 min: Ujjayi pranayama (Victorious breathing): 5 repeats, Anuloma-Viloma/ Nadi Shodhana (Alternate nostril breathing): 10-15 repeats, Alternate Kapalapathi pranayama (Alternate nostril rapid exhalations): 5 repeats, Suryabhedhana pranayama (Right nostril breathing): 5 repeats	Shavasana (Corpse pose/ Deep relaxation): 5 min, One–one meditation: 5 min, Anapanasati (Breath counting meditation): 10 min	-Practice was dapted according to abilities of the participants<br>- Classes had 25 participants<br>-At the end of 2 weeks, advice was given on ongoing medical treatment
Telles, 2013	NR	I1= 10 min<br>I2= 10 min	NR	NA	NA	I1= 10 min: Anuloma-Viloma/Nadi Shodhana (Alternate nostril breathing)<br>I2= 10 min: Breath awareness without	NA	-Purdue pegboard task was completed before and after intervention in all groups

						manipulation of the nostrils		- I1 did not include breath retention
Hagins, 2014	-Based on the primary series of Ashtanga yoga and specifically designed for this study by Eddie Stern (Director of Ashtanga Yoga New York)	I= 55 min/day x 2 days/ week x 12 weeks + 20 min/day x 3 days/ week x 12 weeks (home practice)	-Supervised by yoga instructors, who had a minimum 200-hour training and completed a workshop specific to hypertension<br>-Encouraged to practise at home	-$100 was given at the end of the study<br>-Printed text, photos and a DVD were given for home practice	35 min total: Urdva Hastasana (Upward salute): 3 times, Uttanasana (Standing forward bend): 3 times, Chakravakasana/ Marjaryasana-Bitilasana (Cat-cow pose): 3 times, Tiryaka Bhujangasana (Twisted cobra pose): 3 times, Surya Namaskara A: 5 sets, Surya Namaskara B: 3 sets, Virabhadrasana I-II (Warrior 1 and 2): 3 sets, Padahastasana (Hands to foot pose): 2-8 breaths, Trikonasana (Triangle pose): 4-16 breaths, Utthita Parsvakonasana (Extended side angle pose): 4-16 breaths, Prasarita Padottanasana A/B/C/D (Wide-legged standing forward bend): 2-8 breaths each type, Parsvottanasana (Side Stretch pose): 2-8 breaths, Bharmanasana (Table top pose): 2-8 breaths each, Janu Sirsasana (Head-to-knee forward bend): 2-8 breaths each, Baddha Konasana (Bound Angle/ Butterfly pose): 2-8 breaths, Shalabhasana (Locust pose): 2-8 breaths	10 min total: Seated cross legged, hands clasped behind back, head leaning forward, flexed spine (10 breaths), Seated cross legged, leaning backwards with hands on floor, arching spine, looking up and back with eyes (10 breaths), Ujjayi pranayama with Uddiyana Bandha (Victorious breath with Abdominal lock): 10 breaths, Anuloma-Viloma/Nadi Shodhana (Alternate nostril breathing): 10 breaths each side	5-7 min: Guided meditation focusing on the body and breath, nervous system and mind, 5 min: Shavasana (Corpse pose/ Deep relaxation)	-Average intensity was 3 METs<br>-Modified if needed and chairs and the wall were used to support<br>-Provided positive expectations regarding the potential for the class to lower blood pressure
Patil, 2014a	NR	I= 60 min/day x 6 days/ week x 12 weeks	-Supervised by a yoga instructor	NR	5 min: Sukshma Vyayama (Loosening practices), 15 min: Padahastasana (Hand to Foot Pose), Ardha chakrasana (Half wheel pose), Shashankasana (Rabbit pose), Ardha Ustrasana (Half camel pose), Bhujangasana (Cobra pose), Ardha Salabhasana (Half locust pose), Trikonasana (Triangle pose)	Breathing Practices Hands in and out breathing, Ankle stretch breathing, Straight leg raising breathing, Lumbar stretch breathing, 5 min: Anuloma-Viloma/Nadi Shodhana (Alternate nostril breathing), Brahmari ((Bumblee) Bee breathing)	Cyclic Meditation: 23 min, Bhajans (Devotional chanting): 5 min, Opening prayer (1 min), Closing prayer (1 min)	-Practised in the morning between 06:00 and 07.00
Patil, 2014b	NR	I=60 min/ day x 6 days/ week x 6 weeks	-Supervised by an authorised yoga instructor	NR	5 min: Sukshma Vyayama (Loosening practices), 15 min: Padahastasana (Hand to Foot Pose), Ardha chakrasana (Half wheel pose), Shashankasana (Rabbit pose), Ardha Ustrasana (Half camel pose), Bhujangasana (Cobra pose), Ardha Salabhasana (Half locust pose), Trikonasana (Triangle pose)	Breathing Practices Hands in and out breathing, Ankle stretch breathing, Straight leg raising breathing, Lumbar stretch breathing, 5 min: Anuloma-Viloma/Nadi Shodhana (Alternate	Cyclic Meditation: 23 min, Bhajans (Devotional chanting): 5 min, Opening prayer (1 min), Closing prayer (1 min)	_

						nostril breathing), Brahmari ((Bumblee) Bee breathing)		
Sujatha, 2014	NR	I=45 min/ day x 5 days/week x 12 weeks	-Practised at home<br>-Attended group sessions at a community centre<br>-Supervised	-DVD was given for home practice<br>-Attended group session once in two weeks<br>-Attendance register was maintained	18 min (3 min each): Sukhasana (Easy pose), Vajrasana (Thunderbolt pose), Ardha Matsyendrasana (Half lord of the fishes pose), Bhujangasana (Cobra pose), Urdhva Dhanurasana/ Chakrasana (Wheel pose)	12 min (3 min each): Bhastrika (Bellow breathing), Ujjayi (Victorious breathing), Anuloma-Viloma/Nadi Shodhana (Alternate nostril breathing), Kapalabhati (Rapid exhalations)	Shavasana (Corpse pose/ Deep relaxation), Mindfulness meditation	-Yoga practices were taught in the first week: 120 min/day x 5 days/week x 1 week and then they practised at home<br>-Sessions were held at two time points for flexibility
Patil, 2015	NR	I= 60 min/day x 6 days/ week x 12 weeks	-Supervised by experienced authorised instructors	-Attendance register was maintained	5 min: Sukshma Vyayama (Loosening Practices): Loosening of Fingers, Loosening of Wrist, Shoulder rotation, Ankle stretch/ rotation, Drill walking, 15 min: Utkatasana (Chair pose), Padahastasana (Hand to foot pose), Ardha chakrasana (Half wheel pose), Shashankasana (Rabbit pose), Ardha Ustrasana (Half camel pose), Bhujangasana (Cobra pose), Ardha Salabhasana (Half locust pose), Trikonasana (Triangle pose)	Breathing Practices Hands in and out breathing, Ankle stretch breathing, Straight leg raising breathing, Lumbar stretch breathing, 5 min: Anuloma-Viloma/Nadi Shodhana (Alternate nostril breathing), Brahmari ((Bumblee) Bee breathing)	Cyclic Meditation: 23 min, Bhajans (Devotional chanting): 5 min, Opening prayer (1 min), Closing prayer (1 min)	-Practices were taught for the first 2 weeks and then complete module was practised for the last 10 weeks
Pushpanathan, 2015	Validated by ACYTER, JIPMER, according to the MDNIY guidelines	I=45 min/ day x 3 days/ week x 12 weeks	-At a research centre (JIPMER)<br>-Supervised by yoga therapists<br>-Encouraged to practise at home	-Attendance register was maintained	15 min of asanas; Talasana (Palm tree pose), Ardhakati Chakrasana (Lateral arc pose), Ardha Chakrasan (Half wheel pose), Uttanpadasan (Raised leg pose), Ardha Halasana (Half plough pose), Pawanamuktasana (Wind releasing pose), Makarasana (Crocodile pose), Bhujangasana (Cobra pose), Vajrasana (Thunderbolt pose)	15 min of pranayamas; Chandranadi pranayama (Left nostril breathing), Pranav (Om meditation breathing), Anuloma-Viloma/Nadi Shodhana (Alternate nostril breathing)	10 min: Kayakriya in Shavasan (Dynamic body relaxation), Shavasan with Savitri pranayam (Rhytmic breath in corpse pose)	-Sessions started with yogic counselling and preparatory practices (5 min)
Prakash, 2015	NR	I= 30 min/day x 7 days/ week x 8 weeks	-At hospital<br>-Under supervision	NR	Padmasana (Lotus pose), Makarasana (Crocodile pose)	Suryabhedana (Right nostril breathing), Anuloma-Viloma/Nadi Shodhana (Alternate nostril breathing), Kapalbhati (Rapid Exhalations), Ujjayi (Victorious breathing), Sheetali (Cooling	Shavasana (Corpse pose/Deep relaxation)	-Practised at early morning hours

						breath), Shitkari (Hissing breath), Bhastrika (Bellow breathing), Bhramari ((Bumblee) Bee breathing)		
Sriloy, 2015	NR	I=20 min	-At hospital<br>-Supervised by a qualified yoga ar naturopathy physician with 4 years of therapeutic yoga experience	NA	NA	20 min: Adhama pranayama (Abdominal breathing), Madhyama pranayama (Chest breathing), Adhyama pranayama (Clavicular breathing), Anuloma-Viloma/Nadi Shodhana (Alternate nostril breathing)	NA	_
Thiyagarajan, 2015	-Designed in ACYTER, JIPMER	I= 45 min/day x 3 days/ week x 12 weeks	-In the ACYTER, JIPMER<br>-By the qualified yoga teachers<br>-Encouraged to practise at home	-Attendance register was maintained	10 min: Preparatory practices: Breath–body coordination practices, Sukshma Vyayama (Loosening practices), 14 min: Talasana (Palm tree pose), Ardhakati Chakrasana (Lateral arc pose), Ardha Chakrasana (Half wheel pose), Uttanpadasana (Raised leg pose), Ardha Halasana (Half plough pose), Pavanamuktasana (Wind releasing pose), Sarvangasana (Shoulder stand), Makarasana (Crocodile pose), Bhujangasana (Cobra pose), Dhanurasana (Bow pose), Vajrasana (Thunderbolt pose)	7 min: Chandranadi pranayama (Left nostril breathing), Pranav (Om meditation breathing), Anuloma-Viloma/Nadi Shodhana pranayama (Alternate nostril breathing)	14 min: Kayakriya in Shavasana (Dynamic body relaxation), Shavasan with Savitri pranayama (Rhythmic breath in corpse pose)	_
Cohen, 2016	Forrest Yoga (a form of Hatha yoga)	I1=90 min/ day x 2 days/week x 12 weeks + 90 min/ day x 3 days/week x 8 weeks	-At an offsite studio<br>-Supervised by a certified yoga instructor at the studio<br>-Practised at home	-A DVD of Ana Forrest was given for home practice<br>-Logged in LIMBS study website weekly and recorded their practice<br>-Attendance register was maintained by instructors	25 minutes: Seated side and forward bends, e.g. Parsva Sukhasana (Seated side bend), Janu Sirsasana (Head to knee forward bend), twists, e.g. Ardha Matsyendrasana (Half lord of the fishes pose), hip and shoulder openers, e.g. Baddha Konasana (Bound angle pose/Butterfly pose), Agnistambhasana (Firelog pose). 2-3 minutes: Setu Bandha Sarvangasana (Dynamic/static bridge pose), Adho Mukha Svanasana (Downward facing dog), inversions, e.g. Adho Mukha Vrksasana (Handstand). 40 minutes: Sun salutations B series with standing poses, e.g. Virabhadrasana I (Warrior I pose), Virabhadrasana II	5 – 7 min of pranayama (NS)	Shavasana (Corpse pose/Deep relaxation): 5-7 min	After 12 weeks of semi-private classes, participants attended 2 community classes and did one self-practice per week for the remaining 8 weeks of the program.

					(Warrior II pose), Utthita Trikonasana (Extended triangle pose), Backbends, e.g. Ustrasana (Camel ride/Spinal flex pose), Dhanurasanam (Bow pose), Natarajasana (Dancer pose), Urdhva Dhanurasana/Chakrasana (Wheel pose), Apex Poses. 5-7 minutes: Setu Bandha Sarvangasana (Bridge pose), Adho Mukha Svanasana (Downward facing dog), inversions, e.g. Adho Mukha Vrksasana (Handstand), deep twists, forward bends, e.g. Janu Sirsasana ((Seated) head to knee forward bend), Paschimottanasana (Seated forward bend), side bends, straddle, e.g. Upavistha Konasana (Wide-angle seated forward bend), Prasarita Padottanasana (Wide-legged forward bend), splits, e.g. Hanumanasana (Monkey pose)			
Punita, 2016	-Validated by ACYTER and JIPMER in accordance with MDNIY Guidelines	I= 45 min/day x 3 days/ week x 12 weeks	- At JIMPER research centre<br>-Supervised by a trained ACYTER yoga teacher<br>-Encouraged to practice at home	-Attendance register was maintained by instructors	10 min: Breath–body coordination practices and Sukshma Vyayama (Loosening practices), 10 min: Tadasana (Palm tree pose), Ardhakati Chakrasana (Lateral arc pose), Ardha Chakrasana (Half wheel pose), Uttanpadasana (Raised leg pose) Ardha Halasana (Half plough pose), Pavanmuktasana (Wind releasing pose), Makarasana (Crocodile pose), Bhujangasan (Cobra pose), Vajrasana (Thunderbolt pose)	10 min: Chandranadi pranayama (Left nostril breathing), Pranav (Om meditation breathing), Anuloma-Viloma/Nadi Shodhana (Alternate nostril breathing)	15 min: Kayakriya in shavasan (dynamic body relaxation), Shavasan with Savitri pranayam (rhythmic breath in corpse pose)	-Session started with yogic counselling and brief prayer<br>-About diet and lifestyle modification was talked
Wolff, 2016	-Kundalini yoga (Mediyoga) developed at the Institute for Medical Yoga	I= 15 min x 2/day x 7 days/week x 12 weeks	-Practiced at home<br>-Taught by the three doctors who conducted the study and were familiar/ trained mediyoga instructors	-A CD, a nose plug and a manual were given<br>-Self-reporting of home practice via a diary<br>-Access to sessions via a website which was specifically made for the study and allowed to download to phone	Ustrasana (Camel ride/Spinal flex pose): 4 min	Chandranadi pranayama (Left nostril breathing): 11 min	NA	-Practised just after getting out of bed in the morning and just before going to bed in the evening<br>-Information about yoga practice was given by the doctor during a GP consultation<br>-A common template was

								used during the GP consultations
Roche, 2017	-Based on the yoga practice program for the prevention and management of hypertension developed at the ACYTER	I1=95 min/day x 2 days/ week x 8 weeks	NR	-Attendance register was maintained<br>-Participant experiences and perceptions about their health were taken up	45-50 min: Warm-up exercises, Tala Kriya, Hasta Kona Kriya, Trikonasana (Triangle pose), Meru Asana, Sukhasana (Easy pose), Vakrasana (Twisted pose), Chatush Padasana (Four footed pose), Bhujangasana (Cobra pose)	5 min: Adham pranayama (Belly/ abdominal breathing), Dirga/Mahat pranayama (full yogic breath/three-part breath), Savitri pranayama (Rhytmic breath), Anuloma-Viloma/Nadi Shodhana (Alternate nostril breathing), Chandra pranayama, Bhramari pranayama ((Bumblee) Bee breathing), Pranava Pranayama (Om meditation breathing)	10 - 15 min: Shavasana (Corpse pose/ Deep relaxation), Body scan meditation	-Session ended with a talk about yoga and practical application of mindfulness to bring it into every day (10 min)
	-Based on the yoga practice program for the prevention and management of hypertension developed at the ACYTER	I2= 40 min/day x 2 days/ week x 8 weeks	NR	-Attendance register was maintained<br>-Participant experiences and perceptions about their health were taken up	NA	30 min: Adham pranayama (Belly/ abdominal breathing), Dirga/Mahat pranayama (full yogic breath/three-part breath), Savitri pranayama (Rhytmic breath), Anuloma-Viloma/Nadi Shodhana (Alternate nostril breathing), Chandra pranayama, Bhramari pranayama ((Bumblee) Bee breathing), Pranava Pranayama (Om meditation breathing)	NA	-Session ended with a talk on practical application of pranayama techniques (10 min)
	-Based on some of the techniques included in the "Exercise Without Movement" yoga method of the Himalayan	I3= 50 min/day x 2 days/ week x 8 weeks	NR	-Attendance register was maintained<br>-Participant experiences and perceptions about their health were taken up	NA	Anuloma-Viloma/Nadi Shodhana (Alternate nostril breathing)	Shavasana (Corpse pose/Deep relaxation), Shavayatra (61-point relaxation), Body Scan meditation	-Session ended with a talk about yoga and practical application of mindfulness to bring it into every day (10 min)

	Yoga Tradition							
Shetty, 2017	NR	I=20 min/ day x 7 days/ week x 4 weeks	-At a clinical research centre<br>-Observed by the research team	-Attendance register maintained	NA	20 min (10 min each): Sheetali (Cooling breath), Sheetkari (Hissing breath)	NA	_
Supriya, 2017	NR	I= 60 min/day x 3 days/ week x 52 weeks	-Supervised by certified yoga instructors who had at least 5 years of teaching experience	-Supermarket coupon was given at the end of the study<br>-Attendance register maintained	Chakravakasana/Marjaryasana-Bitilasana (Cat-cow pose), Adho Mukha Svanasana (Downward facing dog), Utthitaashwa Sanchalanasana (High lunge), Uttanasana (Standing forward bend), Matsyendrasana (Spinal twist), Urdhva Hastasana (Upward salute), Utkatasana (Chair pose), Virabhadrasana (Warrior pose), Utthita parsvakonasana (Extended side angle pose), Utthita Trikonasana (Extended triangle pose), Vrksasana (Tree pose), Malasana (Garland pose), Eka Pada Rajakapotasana (One-legged king pigeon pose), Salabhasana (Locust pose), Dandasana (Staff pose), Baddha Konasana (Bound angle pose/ Butterfly pose), Agnistambhasana (Fire log pose), Gomukhasana (Cow face pose), Balasana (Child pose), Setu Bandha Sarvangasana (Dynamic/static bridge pose), Supta padangusthasana (Reclining big toe pose), Ananda Balasana (Happy baby pose), Virasana (Hero pose), Centering in cross-legged position, Paripurna Navasana (Knees bend version of boat pose), Vajrasana (Thunderbolt pose), Sukhasana (Easy pose), Eka Pada Bhekasana (1-leg frog pose), Supta Baddha Konasana (Reclining bound angle pose), Uttitha Hasta Padangustasana (Extended big toe pose), Padangusthasana (Big toe pose), Salabhasana (Locust pose), Tadasana (Mountain pose)	Pranayama (NS)	10-min warm-up and 10 min cool-down that consisted of a breathing and relaxation exercise, Shavasana (Corpse pose/Deep relaxation)	- Classes had ~10 participants<br>-Intensity of practices was determined by instructors and modified according to the needs of participants

Misra, 2018	NR	I1= 15 min/day x 5 days/ week x 6 weeks<br><br>I2= 15 min/day x 5 days/ week x 6 weeks	I1:<br>-1 session at a clinic<br>-By an instructor<br>-At least 4 sessions a week at home<br><br>I2:<br>-At least 5 sessions a week at home<br>-Two examiners checked accuracy of practice	I1:<br>-Self-reporting of home practice via a diary (date and time)<br>-Turned in their logs each week at the clinic<br><br>I2:<br>-DVD/YouTube guidance for home practice<br>-Self-reporting of home practice via a diary (date and time)<br>-Turned in their logs each week at the clinic	NA	I1 and I2:<br>Bhastrika (Bellow breathing): 2 min/30 repetitions, Kapalabhati (Rapid exhalations): 5 min/20 cycles, Anuloma-Viloma/Nadi Shodhana (Alternate nostril breathing): 5 min/20 cycles, Bhramari ((Bumble) Bee breathing): 3 times, Om Singing: 3 times	NA	I1 and I2:<br>-The DVD/YouTube contained a 15-minute guided practice
Cramer, 2018	Developed by an expert panel from various yoga traditions, based on earlier studies and classic yoga texts	I1= 90 min/ day x 1 day/ week x 12 weeks	-Supervised by two yoga instructors<br>-Encouraged to practise every day at home	- No minimum duration was suggested for home practice<br>-Audio recordings and training manuals were given for home practice<br>-Self-reporting of home practice via a diary	45 minutes of asanas: Light mobilisation exercises and work on sitting upright: 10 min, Vyaghrasana (Tiger pose): 4 min, Tadasana Vinyasa (Mountain flow): 8 min, Ardha Surya Namaskar (Half sun salutation): 8 min, Paschimottanasana (Seated forward bend): 4 min, Setu Bandha Sarvangasana (Dynamic/static bridge pose) with intervening Apanasana (Knees to chest pose): 8 min, Supta Parivartanasana (Twist-stretch pose): 5 min	10-15 min: Perception exercise in supine position: 3 min, Deeper breathing and completion of breathing with feet flat on floor: 4 min, Setu Bandha Sarvangasana (dynamic/ static bridge pose) with intervening Apanasana (knees to chest pose):2 min, Anuloma-Viloma/Nadi Shodhana (Alternate nostril breathing): 6 min	Meditation-observe stillness: 3 min, Shavasana (Corpse pose/ Deep relaxation): 10 min	-Sessions started with contemplation (3 min) and philosophical/ theoretical topic (12 min)<br>-Adapted to needs of patients and props were used if necessary<br>-Home practice was explained at the end of the sessions
	Developed by an expert panel from various yoga traditions based on earlie studies, classic yoga texts	I2= 90 min/day x 1 day/ week x 12 weeks	-Supervised by the same two yoga instructors<br>-Encouraged to practise every day at home	- No minimum duration was suggested for home practice<br>-Audio recordings and training manuals were given for home practice	NA	60 min of pranayama and dhyana: Light mobilisation exercises and work on sitting upright:4 min, Perception exercise in supine position:5 min, Deeper breathing with feet flat on floor:5 min, Completion of	Meditation: 2 min, Shavasana (Corpse pose/ Deep relaxation): 8 min,	-Sessions started with contemplation (3 min) and philosophical/ theoretical topic (12 min)<br>-Adapted to needs of patients and

				-Self-reporting of home practice via a diary		breathing with feet flat on floor:5 min, Setu Bandha Sarvangasana (Dynamic/static bridge pose) with intervening Apanasana (Knees to chest pose): 4 min, Work on sitting upright: 6 min, Dirga/Mahat pranayama (Full yogic breath):4 min, Ujjayi (Victorious breathing):11 min, Meditation:2 min, relaxation with appreciation of the effect in supine position:3 min, Supta Parivartanasana (Twist-stretch pose):5 min, Observe, deepen, and complete breathing (2 min)		props were used if necessary<br>-Home practice was explained at the end of the sessions
Ankolekar, 2019	NR	I= 60 min/day x 6 days/ week x 15 days	-At Manipal Academy of Higher Education<br>-Monitored to do yoga except on duty off days	NR	Swastikasana (Auspicious pose), Vajrasana (Thunderbolt pose), Suptavajrasana (Supine thunderbolt pose), Tadasana (Mountain pose), Trikonasana (Triangle pose), Parshwa-konasana ((Extended) Side angle pose), Pawanmuktasana (Wind releasing pose), Bhujangasana (Cobra pose), Salabhasana (Locust pose), Dhanurasana (Bow pose), Prasarita Padottanasana (Wide-legged forward bend pose), Vakrasana (Twisted pose)	Anuloma-Viloma/Nadi Shodhana (Alternate nostril breathing), Suryabhedana (Right nostril breathing), Chandrabhedana (Single nostril breathing), Bhramari ((Bumblee) Bee breathing)	Shavasana (Corpse pose/Deep relaxation), Meditation (NS)	_
Gadgil, 2019	NR	I1=40 min	NR	NA	NA	30 min (5 sets): Kapalabhati (Rapid exhalations) (1 min), Kukriya (Panting dog breath) (1 min), Bhastrika (Bellow breathing) (1min) with one-minute rest in between each pranayama	10 min: Shavasana (Corpse pose/ Deep relaxation)	-Practised in a quiet room<br>-Sitting in Vajrasana (Thunderbolt pose)

	NR	I=54 min	-At hospital	NA	NA	54 min (9 sets):Anuloma-Viloma/Nadi Shodhana (Alternate nostril breathing) (2 min), Pranav (Om meditation breathing) (2 min), Savitri pranayam (Rhytmic breath) (2 min) with 1 min rest between each pranayama	10 min: Shavasana (Corpse pose/ Deep relaxation): 10 min	-Practised in a quiet room<br>-Sitting in Sukhasana (Easy pose)
Ghati, 2020	NR	I= 5 min	-Supervised by a yoga instructor	NA	NA	Bhramari pranayama ((Bumblee) Bee breathing): 5 min, breath rate 4-6/min	NA	-Sitting on an easy pose (Sukhasana)
Fetter, 2020	-Developed by experienced yoga and stretching licensed instructor	I=75 min/ day x 2 days/ week x 12 weeks	-At the clinical research centre<br>-Video classes supervised by the author, who worked in the area of Physical Education	NR	10 times: Chakravakasana/ Marjaryasana-Bitilasana (Cat-cow pose), 4 times: Adho Mukha Svanasana (Downward facing dog), Kumbhakasana/ Phalakasana/Adho Mukha Dandasana/ Santolasana (Plank pose), Urdhva Mukha Svanasana (Upward-facing dog pose), 3 times: Surya Namaskar (Sun salutations): Pranamasana (prayer pose), Urdhva hastasana (Upward salute), Padahastasana (Hand to Foot Pose), Ashwa Sanchalanasana (High lunge pose), Phalakasana/ Adho Mukha Dandasana/ Santolasana (Plank pose), Ashtangasana (Salutation with eight limbs pose), 1 min:Virabhadrasana II (Warrior II pose), 1 min:Parshwa-konasana/ Utthita parsvakonasana (Extended side angle pose), 1 min:Prasarita Padottanasana (Wide-legged forward bend pose), 1 min:Parivrtta Trikonasana (Revolved triangle pose), Vrksasana (Tree pose), Dandayamna Bharmanasana (Balancing table pose), Janu Sirsasana (Head to knee forward bend), 1 min: Ardha Matsyendrasana (Half lord of the fishes pose), 1 min: Baddha Konasana (Bound angle/ Butterfly pose), 1 min: Setu	NA	Shavasana (Corpse pose/ Deep relaxation): 15min, Anapanasati (Breath counting meditation)	-Classes were held in a room equipped with a 32-inch-screen TV between 2:00 and 6:00 pm

					Bandha Sarvangasana (Bridge pose), 1 min: Sarvangasana (Shoulder stand), 1 min: Halasana (Plough pose), 1 min: Matsyasana (Fish pose)			
Sathe, 2020	NR/NA	I=5 min	-Guided through video and audio casettes	NA	NA	Bhramari pranayama ((Bumblee) Bee breathing): 5 min, breath rate 3/min	NA	-Practised in a relaxed position (Sukhasana) with eyes closed
Thanalakshmi, 2020	NR	I= 30 min/ day x 7 days/week x 12 weeks	-Supervised by a qualified yoga and naturopathy doctor	-Attendance register maintained	NA	30 min (20 sets): Sheetali pranayama (Cooling breath), 1 set included 2 min rest following 10 rounds of practice	NA	-Practised between 7:00 to 9:00 a.m. in empty stomach for 4 weeks
Dhungana, 2021	NR	I=30 min/ day x 5 days/ week x 12 weeks	-Supervised by health workers, trained by experienced yoga teachers<br>-Received trainings at health centres<br>-Encouraged to practise five days a week at home	-Particpants visited health centre every month and were able to call yoga instructors, if needed<br>-Video recordings were given for home practice<br>-Attendance register maintained	5 min: Sukshma Vyayama (Loosening practices in sitting position: Toe bending, ankle bending, knee bending, finger bending, wrist bending, elbow bending, shoulder rotation, neck bending up and down, Ardha Baddha Konasana (Half butterfly pose)), 2 min each: Ardhakati Chakrasan " (Lateral arc pose), Vakrasana (Twisted pose)	3 min:Breath awareness breathing, 2 min each: Chandranadi pranayam (Left nostril breathing), Sheetali pranayama (Cooling breath), Anuloma-Viloma/Nadi Shodhana (Alternate nostril breathing), Bhramari pranayama ((Bumblee) Bee breathing)	1 min: Om recitation, 9 min: Yoga Nidra (Yogic sleep)	-Participants attended two-hour yoga training sessions for 5 consecutive days

Effective yoga interventions were coloured in light blue. NR: Not reported, NS: Not specified, NA: Not applicable, MDNIY: Morarji Desai National Institute of Yoga, METs: Metabolic Equivalents

Table S3 Critical appraisal of included studies

**Authors**	**Q1**	**Q2**	**Q3**	**Q4**	**Q5**	**Q6**	**Q7**	**Q8**	**Q9**	**Q10**	**Q11**	**Q12**	**Q13**	**Total %**
Murugesan, 2000	U	U	N	U	NA	U	U	U	U	U	U	U	Y	8
McCaffrey, 2005	N	U	N	U	NA	U	Y	N	N	U	U	U	Y	17
Kettner, 2009	U	U	U	Y	NA	Y	Y	N	N	U	U	Y	Y	42
Mourya, 2009	U	U	N	U	NA	Y	Y	N	N	Y	U	U	Y	33
Saptharishi, 2009	Y	U	N	U	NA	U	U	N	U	U	U	U	Y	17
Khadka, 2010	N	U	N	U	NA	U	Y	U	U	U	U	U	Y	17
Cohen, 2011	U	U	N	N	NA	U	Y	N	N	Y	Y	U	Y	33
Bhavanani, 2012	U	U	U	U	NA	U	U	Y	Y	Y	Y	U	Y	42
Shantakumari, 2012	N	U	N	U	NA	U	Y	Y	Y	U	U	Y	Y	42
Telles, 2013	Y	U	N	U	NA	Y	U	Y	Y	U	U	N	Y	42
Sujatha, 2014	U	U	N	U	NA	U	U	Y	Y	Y	U	U	Y	33
Hagins, 2014	Y	Y	N	N	NA	Y	Y	N	N	Y	Y	Y	Y	67
Patil, 2014a	Y	U	N	N	NA	U	U	N	N	Y	U	U	Y	25
Patil, 2014b	Y	U	Y	N	NA	U	U	U	U	Y	U	U	Y	33
Patil, 2015	Y	U	N	N	NA	U	Y	Y	Y	Y	Y	U	Y	58
Sriloy, 2015	U	U	U	U	NA	Y	U	N	N	Y	Y	U	Y	33
Prakash, 2015	U	U	U	U	NA	U	U	U	U	U	U	U	Y	8
Pushpanathan, 2015	U	Y	U	U	NA	U	Y	N	N	U	U	U	Y	25
Thiyagarajan, 2015	U	Y	Y	N	NA	U	U	N	N	Y	U	U	Y	33
Cohen, 2016	U	U	N	N	NA	N	U	N	Y	Y	Y	Y	Y	42
Punita, 2016	U	Y	N	U	NA	U	Y	N	N	Y	Y	U	Y	42
Wolff, 2016	Y	U	N	U	NA	Y	Y	N	N	Y	Y	Y	Y	58
Roche, 2017	N	U	N	U	NA	U	U	N	N	U	U	U	Y	8
Shetty, 2017	Y	U	N	N	NA	Y	Y	Y	Y	Y	U	U	Y	58
Supriya, 2017	Y	U	U	U	NA	U	Y	N	N	Y	Y	Y	Y	50
Misra, 2018	Y	U	N	U	NA	Y	Y	N	N	U	U	Y	Y	42
Cramer, 2018	Y	Y	N	N	NA	Y	Y	N	Y	U	U	Y	Y	58
Ankolekar, 2019	N	U	U	U	NA	U	U	U	U	Y	U	U	Y	17
Gadgil, 2019	U	U	U	U	NA	U	U	U	U	U	U	U	Y	8
Fetter, 2020	U	U	N	Y	NA	Y	U	N	N	U	U	U	Y	25
Ghati, 2020	Y	U	N	N	NA	U	U	N	N	Y	Y	Y	Y	42
Sathe, 2020	U	U	Y	U	NA	U	U	Y	Y	U	U	U	Y	33
Thanalakshmi, 2020	Y	Y	N	N	NA	Y	U	N	Y	Y	Y	Y	Y	67
Dhungana, 2021	Y	U	N	N	NA	N	U	N	Y	Y	Y	Y	Y	50
Total %	41	18	9	6	0	32	44	21	32	56	35	26	100	

Question 5 was excluded from the denominator and row percentage was calculated out of 12 questions.
This tool uses a series of criteria that can be scored as being met (yes), not met (no), unclear or not applicable (n/a).
Y=yes; N=no; U=unclear; NA=not applicable.
JBI critical appraisal checklist for randomised controlled trials: Q1. Was true randomisation used for assignment of participants to treatment groups? Q2. Was allocation to treatment groups concealed? Q3. Were treatment groups similar at baseline? Q4. Were participants blind to treatment assignment? Q5. Were those delivering treatment blind to treatment assignment? Q6. Were outcomes assessors blind to treatment assignment? Q7. Were treatment groups treated identically other than the intervention of interest? Q8. Was follow-up complete and if not, were differences between groups in terms of their follow up adequately described and analysed? Q9. Were participants analysed in the groups to which they were randomised? Q10. Were outcomes measured in the same way for treatment groups? Q11. Were outcomes measured in a reliable way? Q12. Was appropriate statistical analysis used? Q13. Was the trial design appropriate, and any deviations from the standard RCT design (individual randomisation, parallel groups) accounted for in the conduct and analysis of the trial?

Funnel Plots

Figure S1 Yoga versus control (SBP)

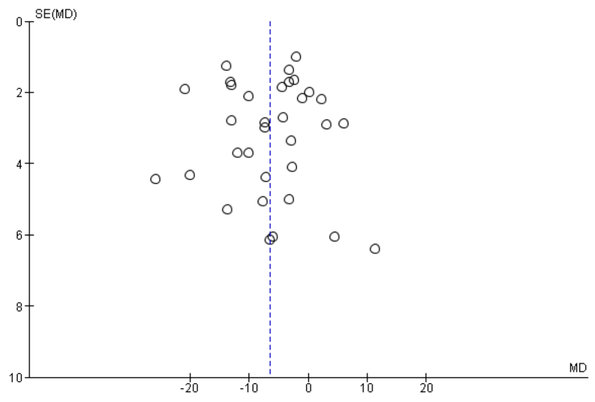

Figure S2 Yoga versus control (DBP)

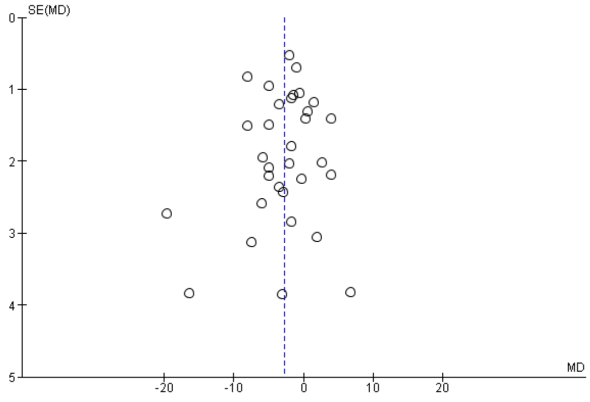

Figure S3 Asana, pranayama and dhyana and relaxation practice versus No intervention (SBP)

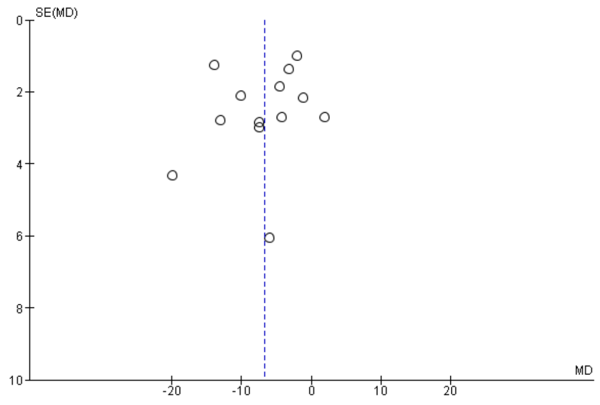

Figure S4 Asana, pranayama, and dhyana and relaxation practice versus No intervention (DBP)

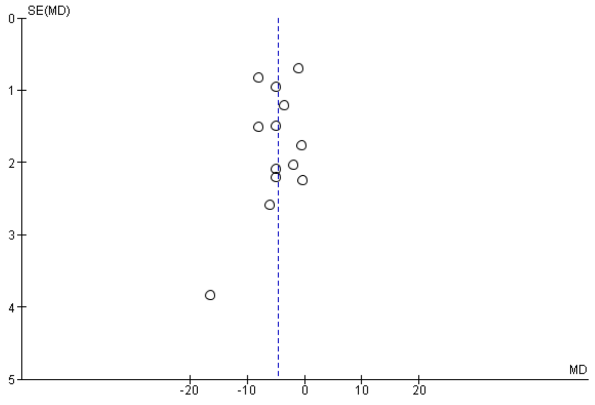

Table S4 Yoga practices used in effective interventions

Asana			Pranayama			Dhyana and relaxation practices		
**Sanskrit Name**	**English Name**	**Frequency of use**	**Sanskrit Name**	**English Name**	**Frequency**	**Sanskrit Name**	**English Name**	**Frequency of use**
Ardha Baddha Konasana	Half butterfly pose		Alternate Kapalapathi Pranayama	Alternate nostril rapid exhalations		Anapanasati	Breath counting meditation	
Ardha Chakrasana	Half wheel pose	x5	Anuloma-Viloma/ Nadi Shodhana Pranayama	Alternate nostril breathing	x14	Kayakriya in Shavasana	Dynamic body relaxation	x3
Ardha Halasana	Half plough pose	x4	Bhastrika Pranayama	Bellow breathing	x4	Shavasana with Savitri Pranayama	Rhythmic breath in corpse pose	x3
Ardha Matsyendrasana	Half lord of the fishes pose	x2	Bhramari Pranayama	(Bumble) Bee breathing)	x6	Shavasana	Corpse pose/Deep relaxation	x9
Ardha Salabhasana	Half locust pose	x3	Chandranadi Pranayama	Left nostril breathing	x4	Vipassana	Mindfulness meditation	
Ardha Ustrasana	Half camel pose	x2	Chandrabhedana Pranayama	Single nostril breathing		Yoga Nidra	Yogic sleep	
Ardhakati Chakrasana	Lateral arc pose	x5	Dirga/Mahat Pranayama	Full yogic breath/ three-part breath			Cyclic meditation	x2
Bhujangasana	Cobra pose	x11	Kapalabhati Pranayama	Rapid exhalations	x4		Opening prayer	x2
Dhanurasana	Bow pose	x4	Kukriya Pranayama	Panting dog breath			Closing prayer	x2
Gomukhasana	Cow face pose		Pranav Pranayama	Om meditation breathing	x4		Om recitation and meditation	x3
Hardhayastambhasana, Navasana/ Naukasana	Boat pose		Savitri Pranayama	Rhythmic breath			One–one meditation	
Janu Sirsasana	Head to knee forward bend pose		Sheetali Pranayama	Cooling breath	x4			
Makarasana	Crocodile pose	x7	Shitkari Pranayama	Hissing breath	x2			
Matsyasana	Fish pose		Suryabhedana Pranayama	Right nostril breathing	x3			
Padahastasana	Hand to foot pose	x2	Ujjayi Pranayama	Victorious breathing	x3			
Padmasana	Lotus pose	x3		Hands in and out breathing	x2			
Parivrtta Trikonasana	Revolved triangle pose			Ankle stretch breathing	x2			
Parshwa-konasana/ Utthita Parsvakonasana	Extended side angle pose			Straight leg raising breathing	x2			

Paschimottanasana	Seated forward bend	
Pawanmuktasana	Wind releasing pose	x6
Prasarita Padottanasana	Wide-legged forward bend pose	
Salabhasana	Locust pose	
Sarvangasana	Shoulder stand	
Shashankasana	Rabbit pose	x4
Sukhasana	Easy pose	
Sukshma Vyayama	Loosening practices	x4
Suptavajrasana	Supine thunderbolt pose	
Swastikasana	Auspicious pose	
Tadasana	Mountain pose	x3
Talasana	Palm tree pose	x3
Tarasana	Star pose	
Trikonasana	Triangle pose	x4
Urdhva Dhanurasana/ Chakrasana	Wheel pose	x3
Ustrasana	Camel ride/spinal flex pose	
Utkatasana	Chair pose	
Uttanpadasana	Raised leg pose	x3
Vajrasana	Thunderbolt pose	x8
Vakrasana	Twisted pose	x4
Viparita Karani	Legs up the wall	
Yoga Mudrasana	Yoga seal pose	x3
Yoni Mudra		

Lumbar stretch breathing	x2
Breath awareness	x2
